# Feasibility and Acceptability of an Electronic Health HIV Prevention Toolkit Intervention With Concordant HIV-Negative, Same-Sex Male Couples on Sexual Agreement Outcomes: Pilot Randomized Controlled Trial

**DOI:** 10.2196/16807

**Published:** 2020-02-11

**Authors:** Jason William Mitchell, Ji-Young Lee, Yanyan Wu, Patrick S Sullivan, Rob Stephenson

**Affiliations:** 1 Office of Public Health Studies Myron B Thompson School of Social Work University of Hawaii Honolulu, HI United States; 2 Department of Public Health Sciences University of Miami Miami, FL United States; 3 Rollins School of Public Health Emory University Atlanta, GA United States; 4 The Center for Sexuality and Health Disparities Department of Systems, Population and Leadership, School of Nursing University of Michigan Ann Arbor, MI United States

**Keywords:** telemedicine, HIV, couples, sexual and gender minorities

## Abstract

**Background:**

There is a need to develop innovative and accessible dyadic interventions that provide male couples with the behavioral skills to manage the risk of HIV transmission within their relationship.

**Objective:**

We conducted a pilot randomized controlled trial (RCT) to assess the feasibility and acceptability of the electronic health (eHealth) HIV prevention toolkit intervention to encourage seroconcordant negative male couples in the United States to establish and adhere to a sexual agreement (SA).

**Methods:**

Eligible, consented couples were randomly assigned to the intervention or education control and followed up for 6 months, with assessments occurring every 3 months after baseline. Acceptability items were assessed at both follow-up assessments. Descriptive and comparative statistics summarized cohort characteristics, relationship dynamics, and SA outcomes for the entire cohort and by trial arm. To examine the association between couples’ relationship dynamics and their establishment of an SA over time and by trial arm, multilevel logistic regression analyses were performed with a random intercept to account for correlations of repeated measurements of relationship dynamics at months 3 and 6; the odds ratio (OR) of establishment of an SA and the corresponding 95% confidence interval were then reported.

**Results:**

Overall, 7959 individuals initiated screening. Reasons for individual ineligibility varied. An electronic algorithm was used to assess couple-level eligibility, which identified 1080 ineligible and 266 eligible dyads. Eligible couples (n=149) were enrolled in the pilot RCT: 68 received the intervention and 81 received the education control. Retention was 71.5% (213/298 partnered men) over the 6 months. Participants reported high acceptability of the intervention along with some areas for improvement. A significantly higher proportion of couples who received the intervention established an SA at 6 months compared with those who received the education control (32/43, 74% vs 27/50, 54%; *P*=.05). The OR of establishing an SA for couples in the intervention versus those in the control condition was greater than 2 when controlling for a number of different relationship dynamics. In addition, the odds of establishing an SA increased by 88% to 322% for each unit increase in a variety of averaged relationship dynamic scores; the opposite result was found for dynamics of stigma. Differences between trial arms for SA type and adherence were nonsignificant at each assessment. However, changes in these 2 SA aspects were noted over time. The average number of items couples included in their SA was 18, and about one-fourth to one-third of couples included HIV prevention items.

**Conclusions:**

The findings demonstrate strong evidence for the acceptability and feasibility of the eHealth toolkit as a brief, stand-alone, couples-based HIV prevention intervention. These findings support the need to update the toolkit and evaluate it in a larger clinical trial powered for efficacy.

**Trial Registration:**

ClinicalTrials.gov NCT02494817; http://clinicaltrials.gov/ct2/show/NCT02494817

## Introduction

### Background

National estimates indicate that between one-third and two-thirds of HIV infections among gay, bisexual, and other men who have sex with men (GBMSM) occur within primary relationships (ie, male couples) [1,2]. In response to these estimates, a growing interest in couples-based approaches to HIV prevention [3-10] has emerged to investigate how relationship dynamics may affect male couples’ risk for HIV and other sexually transmitted infections (STIs) and the development of interventions for this population. There is a need to develop innovative dyadic interventions that provide couples with the behavioral skills to manage the risk of HIV transmission within their relationship.

### Couples Interdependence Theory

Couples interdependence theory (CIT) is a useful health behavior change theory to understand and examine the process in which relationship dynamics (ie, interaction between primary partners) positively and negatively impact male couples’ decisions and behaviors relative to their risk for HIV and other STIs [11,12]. CIT describes one potential process of how relationship partners influence, initiate, and maintain behaviors that impact one another’s health (ie, interdependence) [11,12]. Relative to HIV/STI prevention, this theoretical framework takes into consideration predisposing factors of the couple, which includes their relationship functioning (eg, commitment, satisfaction, and trust), communication style, perceptions of HIV/STIs as a health threat, and preferences for outcomes associated with the health threat (eg, condom use and testing).

In CIT, these predisposing factors are posited to affect couples’ transformation of motivation and communal coping, 2 other key features of CIT. Transformation of motivation refers to the couple’s cognitive interpretation and emotional response to the health threat as being meaningful (ie, important) to their relationship. In other words, partners move from a primarily individual-focused motivation to one that is more prorelationship and health enhancing (ie, how both partners as a couple benefit instead of only one) [11,12]. Transformation of motivation also lends itself to the couple, creating joint goals for long-term relationship functioning, and each partner’s willingness to accommodate for the relationship is a function of the dynamics present in the relationship [13,14].

Another key component to CIT is communal coping, which refers to partners having a shared assessment of HIV/STIs as a health threat, a vision of shared action about managing and reducing their risk for HIV/STIs (via behaviors) and engaging in related HIV/STI prevention behaviors that are beneficial to them as a couple [11,12]. Couples’ coping strategies for HIV/STI prevention are largely determined by the degree that both partners appraise HIV/STIs from an individual standpoint to one as a collective team (ie, transformation of motivation), such that their shared emotional and cognitive responses lead to a greater likelihood of them making a joint effort, partaking in planning and decision making and communicating about how best to reduce their risk for HIV and other STIs [15,16]. CIT provides a useful theoretical framework to examine how couples’ dynamics in general and changes in their dynamics (eg, predisposing factors, transformation of motivation, and communal coping) may lead relationship partners to working together to engage in and achieve their joint health goals as it applies to HIV/STI prevention.

### Sexual Agreements

Sexual agreements (SAs) are one dynamic of male couples’ relationships that have implications for HIV/STI prevention, as supported by a number of investigations identified in a recent scoping view [17]. An SA is formed when partners have explicit conversations with decision making that leads them to having a mutual understanding about which sexual and other relational behaviors, they want to occur with each other (ie, in their relationship) and if applicable, with anyone else (eg, casual sex partners) [18,19]. To date, much research has been conducted about male couples’ SAs, including circumstances and reasons for forming an agreement [18,20-23], investment in one [24-27], and adherence rates and disclosure, and reasons when an agreement is broken [17,28]. SAs are common among male couples [18,20,21,25,29], vary by type [18,19,29,30] and are dynamic, such that changes in composition or type may occur over time [31]. Types of SAs and the composition of these types come in many forms, yet they generally fall into 3 broad categories of closed, open with guidelines, and open without guidelines. A closed agreement represents that sex only occurs between the primary relationship partners, whereas an open agreement with or without guidelines permits certain (or any) sexual and relational behaviors to occur with casual sex partners.

Implicitly, SAs have direct implications for HIV/STI prevention as this dynamic pertains to couples’ sexual behaviors, which may or may not affect their risk for HIV and other STIs. A recent scoping review summarized the associations reported from previous studies on male couples’ establishment and adherence to the agreement and their engagement in condomless anal sex (CAS) within and/or outside of the relationship [17]. In general, negative associations were found between engagement in CAS outside of the relationship and couples who concurred about having an agreement (including type) and to adhering to it [17]. Other work has found that partnered GBMSM’s likelihood of having had CAS within and outside of the relationship significantly decreased as their scores of being invested in the SA increased [32].

The associations between couples’ SAs and their attitudes toward couple’s HIV testing and counseling (CHTC) and pre-exposure prophylaxis (PrEP) [33] has also been explored [34-38]. Findings from these studies point for the need to tailor content and messaging that account for couples’ perceived concerns and benefits about using these prevention strategies relative to how it may affect their relationship and agreement.

By definition and in consideration of CIT, the process of establishing an SA could be advantageous for helping couples to reduce their risk for HIV and other STIs in several ways. First, it may provide couples with opportunities to learn and practice communication and negotiation skills, including the facilitation of discussions about their previous and current behaviors pertinent to prevention (eg, sex and substance use) and ways forward. Second, creation of an SA could help couples foster having a joint responsibility and identify ways for partners to support one another and for them to make associated decisions for how best to prevent HIV and other STIs in their relationship. For instance, creating an SA could enable couples with opportunities to decide if and when to use various evidence-based HIV/STI prevention strategies in their relationship according to their HIV serostatus and relational and sexual needs. Such strategies could include condom use, individual HIV/STI testing, CHTC, PrEP, and/or treatment as prevention (TasP) [39-42] with antiretroviral treatment (ART) to obtain and maintain an undetectable viral load to decrease the risk of onward HIV transmission among those living with HIV. As noted in prior work with male couples [43-49], the strategies which couples could include in their agreement may depend on the support and needs of each partner in the relationship, their attitudes toward these strategies, and their value and engagement in behaviors (eg, CAS and substance use) that may increase their risk for HIV yet be at odds with certain dynamics of their relationship (eg, trust and intimacy).

### Couples-Based HIV Prevention Interventions for Male Couples

One meta-analysis has concluded that couples-based interventions are more effective in promoting sexual risk reduction behaviors and testing for HIV and other STIs when compared with interventions delivered to individual partners [8]. Although the evidence to support this conclusion is tempered by the limited number of efficacious HIV prevention interventions available for male couples [7], several theoretically informed, couples-based HIV prevention interventions have been developed for male couples [50-59], with several pending dissemination of outcome findings [50,51,54,58]. Many of these current and upcoming interventions use a tailored approach to accommodate couples’ specific needs, incorporate communication and other dynamics in relationship skills–building activities (eg, problem solving), provide sexual health education and HIV/STI prevention-related resources, and encourage the formation of an SA or risk-reduction plan.

Several of the interventions include CHTC as one of the core components, either delivered in person [52,53,55,56] or remotely (ie, video Web-based platform) [51,54]. The number of sessions in the interventions vary, from 2 [50,51], 3 [55] and 4 [52,58] up to 7 sessions [59]. CHTC is a single-session intervention [56] that has also been pilot tested with an added component to address substance use [53]. The delivery time for these interventions also varies, ranging from 45 min for a single session (eg, CHTC) up to 10 or more hours for all sessions.

With respect to specific populations of male couples, 2 of the interventions were designed for young GBMSM in relationships [51,52]: one for methamphetamine-using black male couples [59] and another for predominantly Spanish-speaking Latino GBMSM and their same-sex partners [57,58]. Two of the interventions were developed to attend to the HIV care and adherence needs of male couples with one or both partners living with HIV (ie, serodiscordant and seroconcordant positive) [50,55], whereas some focus on the HIV prevention needs of seroconcordant negative and serodiscordant male couples [51,53,56]. Other interventions address the HIV prevention needs of all 3 groups of couples: seroconcordant negative, seroconcordant positive, and serodiscordant [52,58,59].

To date, 2 of the in-progress interventions are being delivered on the Web [51,54], whereas the rest are being or have been provided in person. In-person interventions for male couples may have limited impact and reachability because of structural barriers (eg, stigma of same-sex behaviors and lack of lesbian, gay, bisexual, transgender, and questioning/queer [LGBTQ]-affirming environments) and the number of resources (eg, appropriately trained personnel and cost) required for successful dissemination and implementation [60-64]. Interventions delivered by a digital health platform (ie, mobile health [mHealth] and electronic health [eHealth]) may help negate some of these limitations and required resources. Couples-based HIV prevention interventions that are delivered by a digital health platform would offer male couples the convenience of accessing the intervention from anywhere with an internet connection and being able to use it in a private setting, thereby providing further privacy, security, safety, and confidentiality. Pending the structure of the intervention, digital health platforms could also help increase reachability as more male couples would be able to use the intervention at any given time compared with those offered in person.

### Specific Aims of the Pilot Randomized Controlled Trial of the Electronic Health HIV Prevention Toolkit Intervention

To help increase the number of accessible HIV prevention interventions for male couples in the United States, we leveraged the digital platform of eHealth. The present eHealth, couples-based HIV prevention toolkit intervention, was developed for seroconcordant HIV-negative male couples, theoretically guided by CIT for couples’ health behavior change [11,12], and was based on preliminary work conducted with the target population [29,31,57] and the extant literature [7,28]. The toolkit intervention is an interactive, directed, experiential website aimed to help prepare each couple with the knowledge and skills needed to create a tailored SA that meets the needs of their relationship and for HIV/STI prevention. The specific aims of the pilot randomized controlled trial (RCT) were to (1) assess the feasibility to recruit, enroll, and retain an eligible and consented sample of couples for 6 months; (2) assess the overall acceptability of the toolkit intervention; (3) examine the preliminary impact that using the toolkit intervention will result in a greater proportion of couples to establish and adhere to an SA compared with couples in the control condition; (4) describe the composition of couples’ SAs relative to HIV/STI prevention; and (5) examine which relationship dynamics were associated with couples’ establishment and/or adherence to an SA. The trial was not adequately powered to find meaningful differences between trial arms.

## Methods

### Study Design

**Figure 1:**
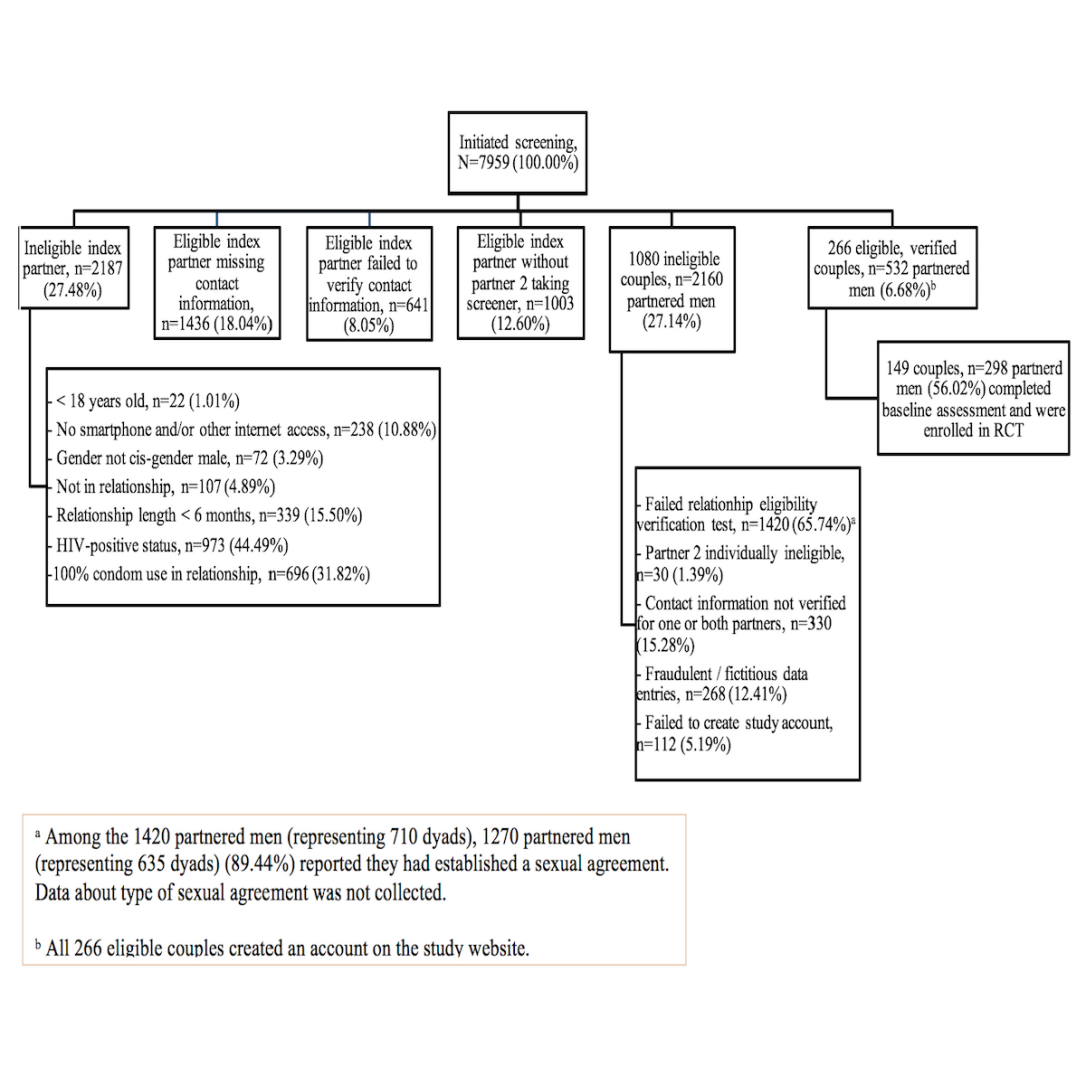
Results of eligibility screening.

All procedures for the pilot RCT occurred on the Web, with couples randomly assigned to 1 of 2 conditions after completion of the baseline assessment. An electronic algorithm was employed to screen and verify couples for study eligibility, followed by manually checking the validity of their data before inviting them to enroll into the pilot RCT. Figure 1 illustrates the Consolidated Standards of Reporting Trials diagram of the RCT. The University of Miami’s institutional review board approved all the study procedures. The pilot RCT was registered on ClinicalTrials.gov (NCT02494817).

### Recruitment and Screening Procedures

Targeted advertisements were placed on Facebook to recruit same-sex male couples over the course of 6 months; findings from these campaigns have been previously described and published (blinded). The advertisements targeted English-speaking adult males living in the United States (≥18 years) who had an interest in men and one of these relationship statuses: married, engaged, domestic partnership, civil union, or in a relationship. Each advertisement included a picture of a male couple with a brief study descriptor and a Web link that led interested individuals to the study introductory website. The study introductory website included webpages for the electronic consent document; eligibility screener; inputting and verifying contact information; and an embedded, electronic algorithm that automatically determined study eligibility at the individual and couple levels. The study introductory website was integrated with SurveyGizmo, a Health Insurance Portability and Accountability Act–compliant Web-based survey tool and database server, to collect and store data for the consent and eligibility screener. On the basis of our prior work leading to this pilot RCT, the electronic algorithm—embedded within the Web-based screener—was developed and used to verify whether both partners of the couple were in a relationship with one another and had met all the eligibility criteria.

After providing consent and completing the screener for individual-level eligibility, potential participants (ie, index partner) were then prompted to provide their own and their partners’ contact information (eg, email and mobile phone); we refer to this participant as the index partner of the couple. At this point in time, the partner of the index partner (ie, partner 2) would receive an email invitation to join the couples-based study that contained a weblink to the study introductory website so he may follow the same procedures for individual-level eligibility, consent, and inputting and verifying contact information. Each individual who provided consent and passed the individual-level eligibility criteria was asked to verify his contact information. Once an individual entered his contact information, he was sent a passcode to his email address and text on his smartphone. He was then asked to enter these passcodes into the study introductory website to verify his contact information.

Once partner 2 completed the same Web-based screening procedures, contact information and screener items from both partners were used to automatically match and evaluate whether they were in a relationship together (ie, couple) and met the additional couple-level eligibility criteria for enrollment in the pilot RCT. This process is described in the following sections.

### Eligibility Criteria

Each partner of the couple—independently—had to meet the following individual-level eligibility criteria to participate in the study: (1) self-report as cis-gender male, (2) aged at least 18 years, (3) be in a current sexual relationship with a main partner for 6 or more months, (4) self-report as HIV negative or unknown serostatus, (5) have had CAS with the primary partner within previous 6 months, (6) self-report no recent history of intimate partner violence or coercion within the previous year, (7) own a smartphone, and (8) have an alternate method to access the internet (eg, computer).

Couples with one or both partners who did not meet one or more of these criteria were individually ineligible for the study and were automatically informed after completion of the electronic screener. For instance, index partners who self-reported living with HIV received a message thanking them for their interest in the study and that they were ineligible to participate; because of being ineligible, his partner (ie, partner 2) would not have received a study invitation by email. The same ineligibility message was emailed to both partners of couples in instances where they were deemed ineligible and/or did not pass the relationship verification test (see the following sections). Thus, in addition to meeting the eligibility criteria, couples also had to pass the couple-level eligibility criteria through verification and validation tests to enroll for the pilot RCT.

### Verification of Couples’ Relationships and Validity of Their Data

After screener data were received from both partners, verification of the couples’ relationship (ie, couple-level eligibility criteria) was done automatically through the electronic algorithm by evaluating and comparing each partner’s response to 5 screener items and using predetermined decisions rules of acceptable responses (see Multimedia Appendix 1). Couples who received a score of 5 on 5 passed the verification test; all other scores were categorized as the couple not passing the verification test. Once a couple was deemed eligible with a verified relationship, we then manually conducted validity checks of their corresponding screener data on a case-by-case basis. Data validity checks consisted of evaluating the following information: repetition of same Internet Protocol (IP) address, use of suspicious participant name(s), presence of duplicate email or fictitious email addresses, back-to-back screener entries, presence of unique data responses to other screener items. For instance, back-to-back screener entries from the same IP address were permitted for a couple as long as all other benchmarks for validation passed. All other instances were flagged as fraudulent and were investigated further by contacting the potential participant/couple for clarification.

### Enrollment and Randomization Procedures for Pilot Trial

All couples had to provide consent, pass eligibility and verification criteria, and post hoc validation tests to enroll into the pilot RCT. Through the electronic screener system, consented, eligible, and verified couples were then randomly assigned a unique enrollment ID containing a 4-digit, 2-letter combination that ended with either .01 or .02 to represent the specific partner in each relationship (eg, 1572SP.01 and 1572SP.02). A 4:4 block allocation was electronically generated and used to randomly assign couples’ enrollment IDs to 1 of 2 eHealth conditions: an information-only control website or the interactive intervention website. Random assignment was double blinded; however, couples may have guessed which condition they were assigned once they completed the baseline assessment and were granted access to the rest of the eHealth website.

Once the ID was assigned, each partner received an email with instructions to log into the study trial website via a computer to create a profile. After creating a study profile, each partner then proceeded to the assigned eHealth study trial website to complete a 45-min baseline assessment electronically. Follow-up assessments occurred 3 and 6 months after baseline and took approximately 45 min to complete. Each participant was sent up to 2 reminders (email and text) about completing each follow-up assessment. Each participant who completed an assessment was compensated with an electronic gift card incentive worth US $25 for his time.

### Description of the Electronic Health HIV Prevention Toolkit Intervention

The toolkit intervention involved participation at both individual and couple levels. At the individual level, participants experienced the interactive website in a directed, sequential fashion before being able to use the website with their relationship partner (ie, couple level). This dual-level intervention design was based on our formative work with 13 same-sex male couples who used the intervention as designed and provided feedback in focus groups (n=7 from Miami, FL, and n=6 from Atlanta, GA), whereas the content and design of the activities and videos were based from our qualitative findings with 29 couples from the metro areas of Detroit, MI, (n=13) and Atlanta, GA, (n=16) [20,22,37]. The vast majority of partnered men from these formative phases stated they wanted an opportunity to read the content, participate in the activities, and have time to digest the material before discussing and comparing their responses to the activities with their relationship partner, including the establish of an SA.

At the individual level, the intervention directed participants through a sequence of instructional and educational videos and modules about evidence-based HIV prevention strategies, communication tips, and SAs. In addition, 3 different activities were also embedded in this series of modules: the creation of a relationship timeline, identification and selection of relationship values, and establishment of an SA via a menu of options arranged by category (see the following sections). After completion of the baseline assessment, each participant was prompted to watch a brief, introductory, 1-min video about the purpose of toolkit intervention and how to use and navigate the website before proceeding to the relationship timeline and value instructional videos and activities. Next, participants were asked to read and review educational content about evidence-based HIV prevention strategies, followed by content on SAs, which included a video that offered suggestions of ways to bring up agreements in the relationship along with some common communication tips (eg, active listening). The last individual-level module was the agreement builder activity with an accompanying instructional video encouraging individuals to begin creating the SA they would like to have with their relationship partner.

Once both partners used the toolkit intervention as directed and added items to their agreement, they were then prompted (via text and email) to sign back into the toolkit intervention website as a couple. Using the toolkit intervention as a couple differed from when participants used it as individuals in important ways. First, the couple were shown their responses to the relationship timeline and value activities in a comparative fashion, which allowed partners to compare their responses and talk about where they differed and how they were similar. These activities served the purpose to *prime* partners to think about the fond memories they created (to date) and what they valued most about being in a relationship with one another collectively, before considering their future via an agreement. Then, the couple was shown a video about constructive communication tips (eg, negotiation) before proceeding to the agreement builder finalization activity. Similar to the other 2 activities, couples could also see—to a degree—how their individual selection of agreement items compared with one another as these pending items were arranged into 3 groupings: definitively wanted, potentially wanted with need to discuss, and did not want with discussion. Couples then negotiated which items they wanted to accept and place into their agreement or reject and place in the trash bin. Once all items were resolved, each partner would finalize his agreement by entering his unique password to the toolkit intervention.

Once a couple finalized their agreement, they could view all content, activities, and videos freely. Furthermore, the interactive website included a searchable resource center database (Sexual Health Resource Center) that allowed participants to find relevant sexual health resources in the United States and the option to download an app of a simplified version of the toolkit intervention that contained a blueprint of the couples finalized agreement, the ability to SMS/text within the relationship (ie, between partners), and the Sexual Health Resource Center. The educational content, videos, and activities were not available on the app.

#### HIV-Prevention Content

This educational module included text that described available evidence-based HIV prevention strategies, including female and male condoms, PrEP, nonoccupational postexposure prophylaxis, individual HIV/STI testing, and couple’s HIV/STI testing.

#### Sexual Agreement Content

Another educational module focused on SAs. The content included an overarching definition of an SA along with different types of agreements that exist within the broader LGBTQ community (eg, closed, open with guidelines, and open without guidelines). Additional text drew from the extant literature about male couples’ SAs to describe how common agreements are among male couples in the United States, what might motivate some to form an SA, the potential benefits of establishing an agreement in the relationship, whether agreements change over time, and the importance of communicating about the agreement in the relationship [17].

#### Relationship Timeline Activity

Participants could choose up to ten milestone life events that occurred throughout their relationship. Some examples of the events on the timeline activity included firsts such as *first kiss, first time I met his family (or he met my family), first big purchase together*. Each event was dated by the participant, which was then automatically placed chronologically in the visual format of a timeline.

#### Relationship Values Activity

Participants could choose up to 5 items that represented what they valued most about in a relationship with their current partner. Some examples of values presented in this activity were *trusting each other*, *commitment to help our relationship grow*, *accepting our differences*, *feeling sexually satisfied with one another*, and *counting on each other*.

#### Agreement Builder Activity

Participants could choose as many items as they wanted in their agreement. A total of 96 items were organized in 5 different categories: wellness (20 items; eg, testing for HIV, eating healthy, and supporting each other in our health goals), social etiquette (9 items; eg, holding hands with partner in public and having profiles on social media websites/apps), sex with my partner (23 items; eg, bottoming without condoms with partner, giving or getting head with partner, and group sex play as a couple), sex with other people (22 items; eg, topping with condoms with others, kissing with others, and sexting with others), and drugs (22 items; eg, alcohol with sex, ecstasy without sex, and erectile dysfunction medications). All 5 categories also included the option for participants to *create their own* and add details to each selected item.

#### Sexual Health Resource Center

This searchable database presented participants with contact and operational information about HIV/STI testing locations throughout the United States by zip code, testing modality preference (eg, individual, CHTC, and over the counter), appointment type (eg, walk-in and appointment required), and cost (eg, free and sliding fee). Locations of pharmacies were also included and searchable by zip code.

### Information-Only Control Condition

Couples assigned to the information-only control condition also received an interactive website that contained the same HIV prevention content and Sexual Health Resource Center, along with access to download a similar app as the intervention group sans the blueprint of an agreement.

### Measures

All participants, regardless of the study arm, were asked to complete the 3-month and 6-month follow-up questionnaires. The content of follow-up surveys matched the content of the baseline survey, except follow-up surveys also collected information on the formation of, type of, and adherence to an SA. In the event that couples ended their relationship, each partner was still asked to complete their participation throughout the 6 months to collect remaining data and receive their incentives. All data from baseline and follow-up assessments were deidentified, anonymized, and stored on secured servers and password-protected computers.

#### Outcome Variables

The present analysis focuses on 2 outcomes: (1) establishment of an SA and (2) adherence to a SA. Data for these outcomes were collected at 3- and 6-month follow-up assessments from all participants, regardless of the study arm.

#### Independent Variables

The baseline assessment captured participants’ demographic (eg, state of residence, age, race, ethnicity, sexual orientation, education, employment, and health insurance regular primary provider) and relationship characteristics (eg, relationship length, type, status, and cohabitation) via categorical or dichotomous responses. A number of common sexual behavior items (eg, CAS by partner type) and measures about HIV/STI testing were also captured.

A variety of relationship dynamics were also assessed by using validated instruments for trust [65], relationship commitment [66], relationship satisfaction [67], relationship sexual satisfaction [68], intimacy [69], communication patterns [70], communal confidence [71], use of communal coping strategies to reduce HIV threat [71], preferences for sexual health outcomes [71], HIV social support scale [72], HIV-negative couples’ perceptions of severity of HIV infection [71], investment in an SA [24], and preferences for general lifestyle outcomes [71]. Perceptions of local stigma [73], perceptions of gay-related stigma [71], and internalized homophobia [74] were also assessed. HIV-related dyadic measures developed for GBMSM in a relationship [71] offer a quantitative way to assess transformation of motivation and communal coping of CIT. All scales were assessed at all 3 time points (baseline, 3 months, and 6 months), except investment in an SA, which was assessed only at the 3- and 6-month time points. Details about the scales used to capture couples’ relationship dynamics are provided in Multimedia Appendix 2.

### Analyses

Descriptive statistics were used to summarize cohort characteristics and relationship dynamic variables for the entire cohort by trial arm and by establishment of SA. Dyadic data were calculated for couples if there were no missing values from either partner.

For continuous variables, couple-level mean variables were generated by taking the averaged value from both partners’ scores, whereas within-dyad variables (couple-level differences) were generated by taking the absolute difference between 2 partners’ scores. Missing values were assigned if either or both partners did not provide a score. Categorical dyadic variables were generated based on whether both partners had the same or different answers. For example, dyadic ethnicity was categorized to 3 levels: both Hispanic, 1 Hispanic, and neither Hispanic. Furthermore, 2-sample *t* tests and chi-square tests were used to evaluate differences between the intervention and control arms for couple-level continuous and categorical independent variables, respectively. To examine the association between relationship dynamics and establishment of an SA, we performed multilevel logistic regression analyses with random intercept for couples to account for correlations of repeated measurements of relationship dynamics at months 3 and 6 and reported the odds ratio (OR) of establishment of an SA and the corresponding 95% confidence interval. All analyses were performed using statistical software R 3.5.2.

## Results

### Aim 1: Feasibility to Screen, Enroll, and Retain an Eligible and Consented Sample

As shown in Figure 1, 7959 individuals initiated screening resulting in 27.48% (2187/7959) of index partners being ineligible at the individual level; the remaining index partners were eligible at the individual level, but 18.04% (1436/7959) did not provide any contact information, 8.05% (641/7959) failed to verify their contact information, and 12.60% (1003/7959) did not have their partners (ie, partner 2) take the screener. The remaining screeners represented both partners of the couple, with 27.13% (2160/7959) being ineligible at the couple level among other reasons. Overall, 532 partners representing 266 couples passed the eligibility, verification, and validation screening process and were invited to participate in the pilot RCT via email invitation. Of these 266 couples, 149 (56.0%) were enrolled in the pilot RCT as indicated in their creation of a required study profile and completion of the baseline assessment.

**Figure 2:**
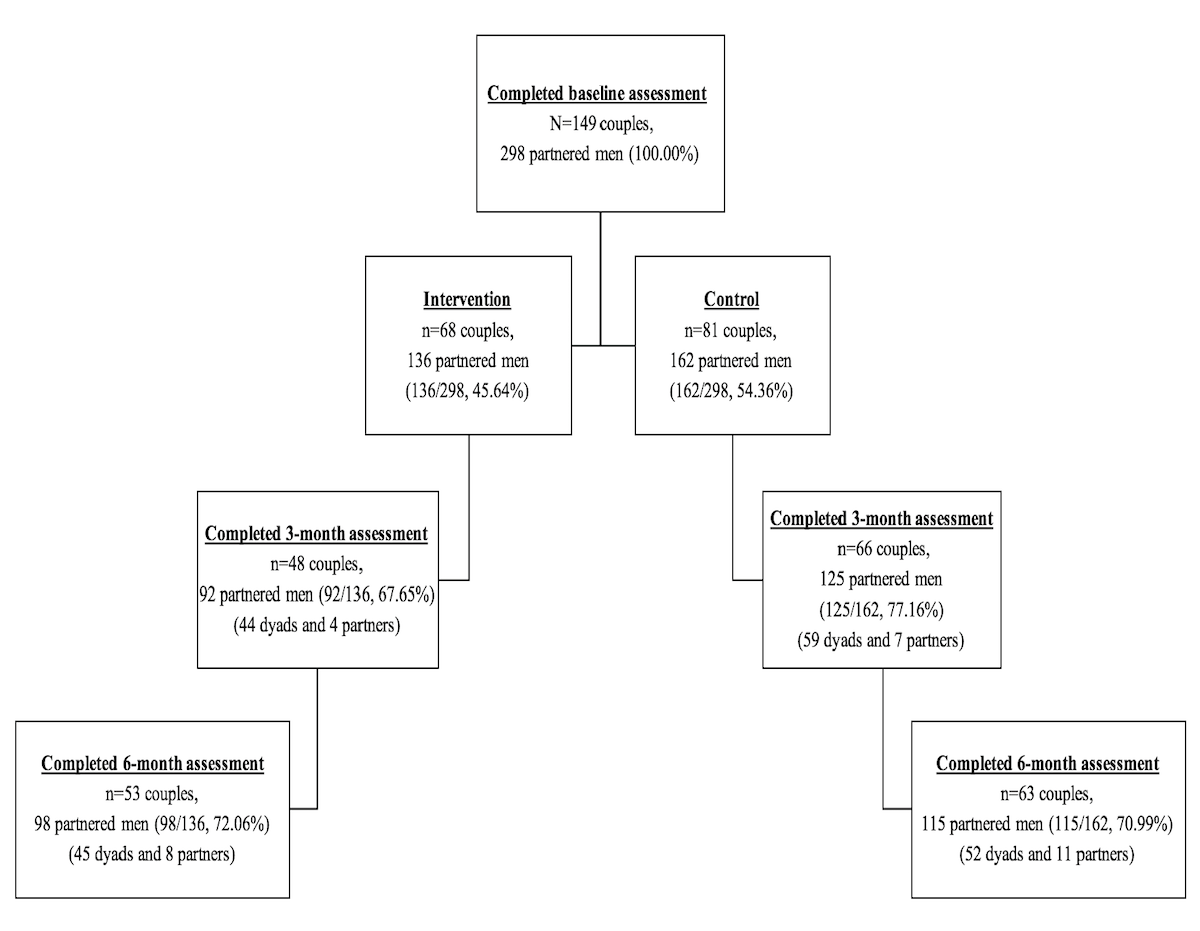
Retention rates of pilot randomized controlled trial.

Figure 2 shows retention rates for the 6-month pilot trial at the individual and couple levels. Overall, 71.5% (213/298) of partnered men were retained at the end of the 6-month pilot trial. Retention rates at the 3-month assessment were 67.6% (92/136) of partnered men in the intervention arm and 77.2% (125/162) of partnered men in the control arm (*P*=.07). Retention rates at the 6-month assessment were 72.1% (98/136) of partnered men in the intervention arm and 71.0% (115/162) of partnered men in the control arm (nonsignificant).

### Sample Characteristics

Baseline characteristics and relationship dynamics for the total cohort and by study arm are provided in Tables 1 and 2, respectively. Given randomization and allocation procedures were used, any differences in baseline characteristics and dynamics are the result of chance rather than bias.

**Table 1:** Cohort baseline demographic and relationship and sexual behavior characteristics by trial arm.

Characteristics			Cohort	Intervention	Control	*P* value
**Couple-level characteristics^a^**						
	**Ethnicity, n (%)**					.64
		Both Hispanic	11 (7.5)	5 (7.5)	6 (7.5)	
		One Hispanic	26 (17.7)	14 (20.9)	12 (15.0)	
		Neither Hispanic	110 (74.8)	48 (71.6)	62 (77.5)	
	**Race, n (%)**					.47
		Both white	121 (81.2)	53 (77.9)	68 (83.9)	
		Multiracial or other race	28 (18.8)	15 (22.1)	13 (16.1)	
	**Sexual orientation, n (%)**					.68
		Both gay	140 (94.0)	65 (95.6)	75 (92.6)	
		1 bisexual, 1 gay	9 (6.0)	3 (4.4)	6 (7.4)	
	**Education attainment, n (%)**					.36
		Both bachelor’s degree or higher	39 (26.5)	18 (26.5)	21 (26.6)	
		One bachelor’s degree or higher	49 (33.3)	19 (27.9)	30 (38.0)	
		Neither have at least bachelor’s degree	59 (40.1)	31 (45.6)	28 (35.4)	
	**Employment status, n (%)**					.84
		Both employed	98 (65.8)	46 (67.7)	52 (64.2)	
		One employed	34 (22.8)	14 (20.6)	20 (24.7)	
		Neither employed	17 (11.4)	8 (11.8)	9 (11.1)	
	**Health insurance, n (%)**					.77
		Both have	112 (75.2)	53 (77.9)	59 (72.8)	
		One has	27 (18.1)	11 (16.2)	16 (19.8)	
		Neither has	10 (6.7)	4 (5.9)	6 (7.4)	
	**Regular general physician/MD, n (%)**					.34
		Both have	85 (57.1)	42 (61.8)	43 (53.1)	
		One has	42 (28.2)	19 (27.9)	23 (28.4)	
		Neither has	22 (14.8)	7 (10.3)	15 (18.5)	
	**US region of residence^b^** **, n (%)**					.16
		Northeast	14 (9.4)	5 (7.3)	9 (11.1)	
		Midwest	40 (26.8)	24 (35.3)	16 (19.8)	
		South	53 (35.6)	20 (29.4)	33 (40.7)	
		West	37 (24.8)	18 (26.5)	19 (23.5)	
		Two regions, long-distance^c^	5 (3.3)	1 (1.5)	4 (4.9)	
	**Relationship type, n (%)**					.14
		Monogamy	130 (87.8)	56 (82.4)	74 (92.5)	
		Open	8 (5.4)	6 (8.8)	2 (2.5)	
		Discrepant reports	10 (7.8)	6 (8.8)	4 (5.0)	
	**Relationship status^d^** **, n (%)**					.08
		Long-term oriented	85 (57.1)	45 (66.2)	40 (49.4)	
		Boyfriends	41 (27.5)	13 (19.1)	28 (34.6)	
		Partners reported differently	23 (15.4)	10 (14.7)	13 (16.1)	
	Relationship length in years, mean (SD)		3.1 (3.66)	3.8 (4.4)	2.6 (2.7)	.04
	Age difference between partners in years, mean (SD)		3.6 (3.9)	2.9 (3.2)	4.1 (4.4)	.07
	**Ever had an HIV test, n (%)**					.82
		Both have	108 (72.5)	51 (75.0)	57 (70.4)	
		One has	34 (22.8)	14 (20.6)	20 (24.7)	
		Neither has	7 (4.7)	3 (4.4)	4 (4.9)	
	**Ever had an STD^e^** **test, n (%)**					.53
		Both have	88 (59.1)	40 (58.8)	48 (59.3)	
		One has	39 (26.2)	20 (29.4)	19 (23.5)	
		Neither has	22 (14.8)	8 (11.8)	14 (17.3)	
	**Has had sex with a casual MSM^f^** **partner in prior 3 months, n (%)**					.11
		Both have	14 (9.4)	10 (14.7)	4 (4.9)	
		One has	39 (26.2)	18 (26.5)	21 (25.9)	
		Neither has	96 (64.4)	40 (58.8)	56 (69.1)	
**Individual-level characteristics^g^**						
	Age (years, range: 18-58), mean (SD)		27.8 (7.16)	28.1 (7.33)	27.6 (7.03)	.49
	**Average number of condomless anal sex episodes with partner in prior 3 months, mean (SD)**					
		Insertive role	8.4 (14.0)	6.3 (11.9)	10.2 (15.4)	.02
		Receptive role	7.9 (13.1)	4.9 (7.7)	10.4 (16.0)	<.001
		Insertive and receptive in same episode	1.7 (5.8)	0.6 (1.7)	2.6 (7.6)	<.01
	Average number of casual MSM partners in prior 3 months (n=68), mean (SD)		3.8 (6.1)	3.8 (6.3)	3.7 (6.0)	.69
	Average number of anal sex episodes with casual MSM partner(s) in prior 3 months (n=67), mean (SD)		0.6 (1.7)	0.3 (0.7)	1.0 (2.5)	.11
	**Average number of condomless anal sex episodes with casual MSM partner(s) in prior 3 months (n=17), mean (SD)**					
		Insertive role	1.4 (2.2)	1.0 (0.7)	1.8 (3.2)	.5
		Receptive role	2.7 (7.2)	0.6 (1.3)	5.1 (10.2)	.2

^a^Cohort, intervention, and control included 149, 68, and 81 couples, respectively.

^b^States and territories not represented: Guam, US Marshall Islands, Alaska, New Mexico, New Hampshire, Maine, Mississippi, and North Dakota.

^c^9 couples were in a long-distance relationship, 4 of whom resided in states within the same US region, whereas 5 couples had partners living in states in 2 different regions (Colorado and Illinois, Florida and Massachusetts, Michigan and Washington, Pennsylvania and Virginia, and Pennsylvania and Wisconsin).

^d^Long-term oriented was classified as couples who had both partners self-reporting one of the following: being married, engaged, had a commitment ceremony, or in a domestic partnership. Boyfriend category included couples who had both partners self-reporting as boyfriends, in a relationship, or none of the above. Discrepant reports represented couples in which one partner reported an option within the long-term oriented classification and the other partner reported an option within the boyfriend classification.

^e^STD: sexually transmitted disease.

^f^MSM: men who have sex with men.

^g^Cohort, intervention, and control included 298, 136, and 162 men, respectively.

**Table 2:** Cohort baseline relationship dynamics by trial arm.

Relationship dynamic		Cohort (298 men, 149 couples)	Intervention (136 men, 68 couples)	Control (162 men, 81 couples)	*P* value
**Dyadic trust scale, mean (SD)**					
	Individual score	4.27 (0.76)	4.28 (0.74)	4.25 (0.77)	.74
	Score difference between partners	0.61 (0.61)	0.64 (0.59)	0.59 (0.63)	.68
**Investment model scale for relationship commitment, mean (SD)**					
	Individual score	5.13 (0.80)	5.15 (0.81)	5.11 (0.79)	.68
	Score difference between partners	0.71 (0.68)	0.68 (0.67)	0.74 (0.68)	.59
**Relationship satisfaction, mean (SD)**					
	Individual score	4.34 (0.73)	4.29 (0.74)	4.38 (0.71)	.29
	Score difference between partners	0.57 (0.58)	0.60 (0.56)	0.55 (0.60)	.61
**Miller social intimacy scale, mean (SD)**					
	Individual score	8.63 (1.07)	8.63 (1.06)	8.62 (1.08)	.96
	Score difference between partners	0.90 (0.97)	0.88 (0.89)	0.92 (1.04)	.77
**Avoidance and withdrawal communication pattern, mean (SD)**					
	Individual score	3.60 (1.53)	3.63 (1.50)	3.57 (1.55)	.73
	Score difference between partners	1.19 (0.91)	1.08 (0.91)	1.29 (0.90)	.16
**Constructive communication pattern, mean (SD)**					
	Individual score	6.57 (1.82)	6.34 (1.90)	6.77 (1.73)	.04
	Score difference between partners	1.56 (1.28)	1.59 (1.25)	1.53 (1.32)	.78
**Couple’s communal confidence, mean (SD)**					
	Individual score	27.60 (5.00)	27.53 (5.09)	27.66 (4.93)	.82
	Score difference between partners	3.68 (3.22)	3.82 (3.20)	3.57 (3.25)	.63
**Communal coping strategies to reduce HIV threat, mean (SD)**					
	Individual score	4.06 (0.89)	4.10 (0.83)	4.02 (0.94)	.40
	Score difference between partners	0.90 (0.81)	0.76 (0.68)	1.01 (0.89)	.07
**Preferences for general lifestyle outcomes, mean (SD)**					
	Individual score	23.08 (3.67)	23.24 (3.87)	22.95 (3.51)	.38
	Score difference between partners	3.16 (2.53)	3.28 (2.66)	3.06 (2.42)	.60
**Preferences for sexual health outcomes, mean (SD)**					
	Individual score	31.61 (4.33)	31.66 (4.05)	31.57 (4.57)	.87
	Score difference between partners	4.22 (4.40)	3.79 (4.01)	4.58 (4.69)	.28
**HIV social support scale, mean (SD)**					
	Individual score	3.32 (0.38)	3.35 (0.36)	3.29 (0.40)	.22
	Score difference between partners	0.38 (0.30)	0.39 (0.30)	0.37 (0.30)	.73
**HIV-negative couples’ perceptions of severity of HIV infection, mean (SD)**					
	Individual score	3.78 (0.76)	3.79 (0.73)	3.76 (0.78)	.75
	Score difference between partners	0.71 (0.55)	0.68 (0.53)	0.74 (0.56)	.51
**Sexual satisfaction with the relationship, mean (SD)**					
	Individual score	3.78 (0.89)	3.69 (0.91)	3.85 (0.87)	.12
	Score difference between partners	0.69 (0.63)	0.78 (0.76)	0.61 (0.49)	.09
**Perceptions of local stigma, mean (SD)**					
	Individual score	3.91 (0.94)	3.92 (0.99)	3.91 (0.90)	.93
	Score difference between partners	0.82 (0.65)	0.78 (0.65)	0.85 (0.65)	.51
**Perceptions of gay-related stigma, mean (SD)**					
	Individual score	4.18 (0.66)	4.27 (0.63)	4.10 (0.67)	.03
	Score difference between partners	0.56 (0.47)	0.52 (0.51)	0.59 (0.43)	.34
**Internalized homophobia, mean (SD)**					
	Individual score	1.66 (0.54)	1.68 (0.60)	1.64 (0.49)	.50
	Score difference between partners	0.51 (0.48)	0.50 (0.51)	0.52 (0.45)	.82

### Aim 2: Use and Acceptability of Toolkit Intervention

Over the period of 6 months, participants in the intervention arm logged into their eHealth toolkit an average of 13.42 times (range 1-38) compared with participants in the control arm who used their information-only website an average of 4.48 times (range 1-23). In total, 64.1% (191/298) participants downloaded the accompanying study app onto their smartphone: 65.4% (89/136) participants in the intervention arm (89 men representing 63 couples) and 63.0% (102/162) of participants in the control arm (102 men representing 74 couples). Differences were noted by arm with respect to whether one or both partners of the couple downloaded the app onto their smartphone. Specifically, a higher proportion of couples in the intervention arm (26/63 dyads, 41%) had both partners download the app compared with those in the control arm (28/74 dyads, 38%).

With respect to the acceptability of the eHealth HIV prevention toolkit, participants in the intervention arm also provided data about their perceptions of how easy it was to use various components of it, ranging from navigating the interactive website to using the agreement builder activity (Table 3). Participants reported, on average, that using different aspects of the intervention was *easy* for most items assessed across both time points. They also perceived downloading the accompanying smartphone app and using the Sexual Health Resource Center on the app was slightly less than *easy*, falling somewhere between *neither difficult nor easy* and *easy* across both time points.

Participants further reported how often they thought they would use an activity like the agreement builder with their partner in their relationship. As shown in Table 3, their responses varied at both assessment time points. About 38.0% (38/100) of participants thought they would use this type of activity on an as-needed basis, whereas between 28% and 32% of participants reported they would use this type of activity at a regular interval (ie, every 3-4 months, every 6 months, or yearly) in the relationship with their partner. In contrast, between 19% to 26% of the participants were not sure about how often they would use this type of activity, and about 8% of participants chose never.

**Table 3:** Acceptability data among participants in the intervention arm, by assessment time point.

Acceptability item		3-month assessment	6-month assessment
**Item stem: How easy was it for you to..., mean (SD)**			
	Navigate the different sections of the toolkit website?	4.10 (0.91)	3.94 (1.04)
	Use the sexual health center on the toolkit website?	4.04 (0.84)	3.93 (0.97)
	Download the toolkit app onto your smartphone?	3.42 (1.03)	3.49 (1.23)
	Use the Sexual Health Resource Center on your smartphone app?	3.44 (1.03)	3.53 (1.15)
	Use the agreement builder activity—by yourself—to identify what items you wanted in an agreement with your partner?	3.94 (0.95)	4.05 (0.94)
	Negotiate and finalize the items you wanted in the agreement with your partner?	3.88 (1.01)	3.90 (1.07)
**Item: Now that you have experienced the agreement builder activity, how often do you think you would use this type of activity while in your current relationship with [partner’s first name/nickname]?, n (%)**			
	Every 3-4 months	18 (18.0)	9 (8.7)
	Every 6 months	8 (8.0)	15 (14.4)
	Every 12 months	2 (2.0)	9 (8.7)
	On as-needed basis	38 (38.0)	40 (38.5)
	I’m not sure	26 (26.0)	19 (18.3)
	Never	8 (8.0)	12 (11.5)
**Item asked at 6 months: Please share any suggestions and/or thoughts that you may have about your experience of using the toolkit intervention. (Participant age, US state of residence, relationship length, agreement type), n**			
	“Surveys were quite lengthy” (27, CT, 3.3 years, closed agreement)	—^a^	23
	“This helped me understand my relationship better. Going through the toolkit every few months made me realize how much things change in relationships over the course of six months.” (20, IN, 6 months, closed agreement)	—	21
	“Too many agreement items… felt overwhelmed by the choices.” (30, CA, 3.2 years, open agreement)	—	17
	“My partner and I liked the idea of the toolkit, but we weren’t sure how often we would use it. It would be nice to have more to do [with it] over time.” (39, TN, 4.3 years, open agreement)	—	14
	“Since the last time I used this, me and my partner’s relationship has gotten stronger and I believe by reading these questions and answering them has helped us communicate and work on building a brighter future for each other. So I want to say thank u so very much.” (29, OR, 4.6 years, closed agreement)	—	12

^a^Not applicable.

### Aim 3: Establishment, Type, and Adherence to a Sexual Agreement

Table 4 provides data about the proportion of couples who established an SA, the type of agreement formed, and whether they adhered to the agreement by trial assessment time point (ie, at 3 and 6 months). Among couples who had both partners provide data, almost two-thirds (63.4%) had established an SA at the 3-month assessment, with a nonsignificantly higher proportion of couples in the intervention arm (29/42, 69%) forming one compared with those in the control arm (35/59, 59%; *P*=.40). At the 6-month assessment, 63.4% of couples had established an SA, with a significantly higher proportion of couples in the intervention arm (32/43, 74%) forming one compared with those in the control arm (27/50, 54%; *P*<.05). In each arm at both time point assessments, the remaining proportion of couples did not establish an SA.

For both assessment time points, a nonsignificantly higher proportion of couples in the control arm reported having a closed agreement than couples in the intervention arm (3 month: 33/35, 94% vs 21/29, 72%, *P*=.07; 6 months: 24/27, 89% vs 26/32, 81%, *P*=.87). In contrast, a nonsignificantly higher proportion of couples in the intervention arm reported having an open agreement containing guidelines than those in the control arm (3 months: 4/29, 14% vs 1/34, 3%, *P*=.07; 6 month: 2/32, 6% vs 1/27, 4%, *P*=.87). Similarly, a nonsignificantly higher proportion of couples in the intervention arm had partners who disagreed about their agreement type than those in the control arm (3 months: 4/29, 14% vs 1/34, 3%, *P*=.07; 6 months: 4/32, 13% vs 2/27, 7%, *P*=.87). Although couples’ type of agreement did not significantly differ by trial arm at either assessment time point, there was a trend at the 3-month assessment, with more couples in the intervention arm having formed an open SA with guidelines compared with those in the control condition (*P*=.07).

**Table 4:** Couples’ establishment, type, and adherence to a sexual agreement by trial arm and assessment time point.

Aspect of sexual agreement		3-month assessment				6-month assessment			
		Intervention, n (%)	Control, n (%)	Difference, %	*P* value	Intervention, n (%)	Control, n (%)	Difference, %	*P* value
**Establishment**		42 (100)	59 (100)	—^a^	.4	43 (100)	50 (100)	—	.05
	Yes	29 (69)	35 (59)	9.8		32 (74)	27 (54)	20.4	
	No/did not concur	13 (31)	24 (41)	−9.8		11 (26)	23 (46)	−20.4	
**Type**		29 (100)	35 (100)	—	.07	32 (100)	27 (100)	—	.87
	Closed	21 (72)	33 (94)	−21.9		26 (81)	24 (89)	−7.6	
	Open with guidelines	4 (14)	1 (3)	10.9		2 (6)	1 (4)	2.6	
	Disagreed about type	4 (14)	1 (3)	10.9		4 (13)	2 (7)	5.1	
**Adherence**		29 (100)	34 (100)	—	>.99	32 (100)	27 (100)	—	.4
	Yes, by both partners	27 (93)	31 (91)	1.9		30 (94)	23 (85)	8.6	
	No, by at least one partner	2 (7)	3 (9)	−1.9		2 (6)	4 (15)	−8.6	

^a^

Among couples who established an SA with both partners providing data, 92.1% had adhered to their agreement at the 3-month assessment, with a slightly nonsignificantly higher proportion of couples adhering to theirs in the intervention arm (27/29, 93%) compared with those in the control arm (31/34, 91%; *P*>.99). At the 6-month assessment, a nonsignificantly higher proportion of couples in the intervention arm self-reported adhering to their agreement (30/32, 94%) compared with those in the control arm (23/27, 85%; *P*=.40). The remaining proportion of couples, in each arm at both time point assessments self-reported not adhering to their agreement.

### Aim 4: Composition and Investment in the Sexual Agreement

When using the agreement builder exercise, couples in the intervention arm, on average, included 18 items in their agreement (range 3-56). The types of items couples had in their agreement varied (Table 5). Overall, couples added more items about wellness than any other category; in contrast, items about drug use were the least included. With respect to HIV prevention, which included items in the wellness, sex with partner, and sex with others categories, 38% (11/29) of couples included regular testing of STIs; 28% (8/29) for regular testing of HIV; 31% (9/29) for topping without condoms with partner; 45% (13/29) for bottoming without condoms with partner; and 28% (8/29) specified sex or no sex with other/casual men who have sex with men partners.

**Table 5:** Couples’ average and range of number of items included in their sexual agreements by agreement category.

Item included	Wellness	Sex with partner	Sex with others	Social etiquette	Drugs
Average number of items	9.24	4.58	0.97	2.06	1.21
Range of number of items	3-17	0-17	0-13	0-8	0-5

A number of couples also included items aimed at strengthening and affirming their relationship; these items were located in the wellness and social etiquette categories of the agreement builder. Specifically, 76% (22/29) of couples included talking to/listening to each other; 66% (19/29) had sharing hobbies; 93% (27/29) for going on dates together; 93% (24/26) for going on vacations together; 45% (13/29) included career/education/job support; and 55% (16/29) had being affectionate with partner in public, holding hands in public, and/or had public recognition of relationship.

In addition, many of the couples included items about health-promotive behaviors. For example, 86% (25/29) of couples included exercising more; 86% (25/29) had eating healthier; 76% (22/29) for managing stress; 31% (9/29) had medical, dental, and eye check-ups; and 55% (16/29) included supporting each other in their health goals.

Multimedia Appendix 3 provides data about participants’ investment in the SA and within-dyad score differences for Sexual Agreement Investment Scale (SAIS) [24]. On average, participants for the entire cohort and by study arm were between *very* and *extremely* invested in the SA they created with their relationship partner. Participants were also committed to it, satisfied with it, and valued the SA as noted by their averaged scores. No significant differences for SAIS were found for individual and within-dyad scores between the 2 trial arms.

### Aim 5: Odds Ratio of Establishing a Sexual Agreement Relative to Couples’ Relationship Dynamics

Multimedia Appendix 4 describes results from multilevel logistic regression that modeled the OR that couples established an SA via self-reported averaged relationship dynamic scores (ie, couples’ mean and absolute mean difference between partner’s scores) adjusting for months of assessment and trial arm. Given the exploratory nature of the pilot RCT, we used a *P* value of .10 and less to detect whether a potentially meaningful (ie, a signal) difference was noted between the 2 trial arms for predicting couples’ establishment of an SA over time.

After controlling for averaged couple score of constructive communication, the OR of establishing an SA for couples in the intervention group versus couples in the control group was 2.33 (95% CI 0.86-6.31; *P*=.09). Similar results were found when controlling for averaged couple scores of preferences for sexual health outcomes (OR 2.23, 95% CI 0.85-5.89; *P*=.10), perceived gay-related stigma (OR 2.53, 95% CI 0.95-6.75; *P*=.06), and internalized homophobia (OR 2.26, 95% CI 0.84-6.10; *P*=.10).

When controlling for averaged within-dyad score for relationship commitment, the OR of establishing an SA for couples in the intervention group versus couples in the control group was 2.24 (95% CI 0.84-5.97; *P*=.10). Similar ORs for intervention group versus the control group were found when controlling for within-dyad score differences of sexual satisfaction (OR 2.31, 95% CI 0.86-6.16; *P*=.09), social intimacy (OR 2.30, 95% CI 0.87-6.10; *P*=.09), avoidance and withdrawal communication pattern (OR 2.34, 95% CI 0.84-6.50; *P*=.10), constructive communication pattern (OR 2.30, 95% CI 0.86-6.16; *P*=.10), communal coping strategies to reduce HIV threat (OR 2.26, 95% CI 0.84-6.10; *P*=.10), preferences for sexual health outcomes (OR 2.34, 95% CI 0.88-6.24; *P*=.09), HIV social support (OR 2.31, 95% CI 0.85-6.26; *P*=.09), perceived local stigma (OR 2.40, 95% CI 0.89-6.47; *P*=.08), perceived gay-related stigma (OR 2.38, 95% CI 0.88-6.43; *P*=.09), and internalized homophobia (OR 2.33, 95% CI 0.88-6.20; *P*=.09).

When controlling for trial group assignment, the odds of establishing an SA increased by 101% for each unit increase in couples averaged dyadic trust score (OR 2.01, 95% CI 0.94-4.32; *P*=.07). Similar results were found when controlling for trial group assignment for couples averaged scores of relationship satisfaction (OR 3.08, 95% CI 1.45-6.55; *P*<.01), social intimacy (OR 1.88, 95% CI 1.07-3.32; *P*=.03), constructive communication (OR 1.46, 95% CI 1.08-1.96; *P*=.01), communal confidence (OR 1.13, 95% CI 1.01-1.26; *P*=.03), communal coping strategies to reduce HIV threat (OR 4.22, 95% CI 2.04-8.73; *P*<.001), and perceptions of severity of HIV infection (OR 1.91, 95% CI 0.95-3.83; *P*=.07). In addition, the odds of establishing an SA decreased by 79% for each unit increase in couples averaged perceived local stigma score (OR 0.21, 95% CI 0.11-0.43; *P*<.001) after controlling for trial group assignment; a similar result was also found for perceived gay-related stigma (OR 0.35, 95% CI 0.16-0.79; *P*=.01).

### Aim 5 Differences in Couples’ Relationship Dynamics Relative to Adherence to a Sexual Agreement Over Time

The majority of couples adhered to their SA at 3- and 6-month assessments (see Table 4). Small sample sizes of nonadherence to an SA inhibit our ability to meaningfully assess whether relationship dynamics were associated with this outcome over time and by trial arm.

## Discussion

The findings from this pilot RCT suggest the feasibility and acceptability of an eHealth HIV prevention toolkit intervention to encourage establishment and adherence to an SA among seroconcordant negative male couples.

### Feasibility

A little more than half of couples (149/266 dyads, 56.0%) who could have enrolled did enroll by following the required steps (ie, create a profile on the study website and complete the baseline assessment). It is possible some men may have found these steps to be cumbersome and/or changed their minds about participating after the eligibility and consent portions of the study. In addition, it is also possible that the decision to participate in the study may be linked to relationship dynamics: those with poor communication may opt to not enroll in a study for male couples. For a future trial, modified enrollment steps could be used to simplify the procedures and to help increase the likelihood of couples following through with the necessary components to participate in the trial. First, a Zoom or phone meeting might help inform eligible participants of what is involved for participating in the trial and lead to higher follow-through rates of enrollment. This added step of enrollment has been implemented in an mHealth HIV testing RCT with GBMSM and has led to higher enrollment rates [75]. Second, the added step of requiring participants to create a user profile for the toolkit could be shortened by using data collected from their responses to the eligibility screener and consent. Specifically, the study website portal could automatically generate a user profile for each partner in an eligible, consented, verified male couple. This change would allow men to complete less information, take less time, and simplify the process by having them choose which contact information method they would like to verify (email address or text for mobile number vs both) and a security question to allow them to reset their password.

Some men also reported that the assessments were too time consuming. It is further possible that participants may have also perceived the compensation to be inadequate for the time it required for them to complete each assessment. These possibilities may help explain the retention rates observed for the pilot trial. Several improvements could be made for a future trial. Future assessments could be shortened by preventing overlap of measures across scales. For instance, the Relationship Satisfaction scale [67] could be eliminated as the Relationship Satisfaction subscale in the Investment Model [66] captures similar information about this dynamic. A subscale, instead of the complete scale, could also be used if it aligns with the theoretical framework of the intervention and overall study. For example, to capture and assess changes in mutual constructive communication patterns over time (3 items), this subscale could be used instead of the entire Communication Patterns Scale (3+8 items) inclusive of the Avoidance and Withdrawal subscale, thereby eliminating 8 questions [70]. As smartphone use continues to increase among the general US population [76], the likelihood that future participants will take their assessments on a smartphone will increase. As such, it will become increasingly important to reduce the amount of time it takes for participants to complete an assessment (eg, from 45 to 20 min) to help increase retention rates. Moreover, a larger study could provide more funds to compensate participants for their time (eg, US $40 vs US $25), which may also help with improving retention. These changes, collectively, could help improve the feasibility of a future, larger RCT of this intervention as other procedures were found to be acceptable without issue (eg, randomization using block allocation, double blinded).

### Acceptability and Use

Overall, participants reported high acceptability of the toolkit intervention. This was particularly true for navigating and using the different components of the interactive website and for individually selecting and then negotiating and finalizing the creation of an SA with their relationship partner. Their acceptability was slightly lower for downloading the accompanying smartphone app and using the Sexual Health Resource Center on the app. It is possible that participants may have had connectivity issues in downloading and/or while using the app, thereby influencing their attitudes toward this part of the toolkit. It is also possible that participants may have perceived the app to be too simplistic and questioned the need for it given the stark contrast of what the app offered compared with the interactive website. Moreover, the items used to assess participants’ acceptability of this couples-based intervention may not have captured all key elements and/or their attitudes about it. Future digital health, couples-based interventions may want to consider using the Health Information Technology Usability Evaluation Scale, a customizable usability evaluation instrument that includes subscales of impact, perceived usefulness, ease of use, and user control [77].

Furthermore, a future iteration of this couples-based HIV/STI prevention intervention toolkit may need to be offered in a variety of formats to further increase reach, access, and acceptability among the target population. It is possible that some of the eligible and consented couples who chose not to enroll (eg, 117/266), did so because they realized the intervention could only be used on a laptop of desktop computer as it was not optimized for smaller screen devices. Moving forward, the toolkit may need to be delivered on a single responsive website that would work across all types of internet-connected devices, including smartphones, tablets, laptops, and desktop computers. It could also be offered on an app for smartphones and tablets, which would allow researchers to send reminders and notifications directly to participants (eg, time to complete an assessment). Future research with the target population is warranted to help decide whether one or both of these options for the next iteration of the toolkit ought to be offered.

Acceptability of the agreement builder activity must also be considered. Overall, participants liked the agreement builder activity and how they experienced and used it (solo followed by as a couple). They also provided feedback about how often they thought they would use it over time. About one-third of the participants thought they would use this activity on a regular basis (ie, at some interval), one-third of them perceived they would use it on an as-needed basis, and one-quarter of them were unsure; few of them said they would never use this type of activity. Similar to the importance of being tested for HIV/STIs at a regular interval (eg, every 3, 6, or 12 months), we believe using the agreement builder activity at a regular interval would be beneficial for the couple. SAs are fluid and could change over time to reflect partners’ and couples’ evolving needs. This type of activity would allow couples to revisit and change their agreement, and it would also provide couples with opportunities to help improve their understanding about behaviors they wish to agree to engage in and not engage in (ie, within-couple concordance). Findings from a recent study with male couples from Boston, Atlanta, and Chicago support this idea. The authors reported weak-to-moderate concordance on couples’ agreements guidelines that pertained to having sex outside of the relationship and for specific sexual behaviors they allowed or disallowed to occur [78]. Although we do not think couples ought to be forced into these types of conversations, a toolkit could be programmed to periodically check in with each partner of the couple to assess their overall satisfaction with the agreement and whether their sexual health and relationship needs have changed from when they first created their agreement or from their last check-in. A future version of this activity could provide this kind of check-in mechanism, either preprogrammed or by a time interval (eg, quarterly) set by both partners of the couple.

Participants mentioned another area of the SA builder activity that warrants attention. Some perceived the agreement builder activity contained too many items for them to consider for their SA (Table 3). In addition, approximately one-quarter to one-third of couples included HIV/STI prevention items in their SA (Table 5), and 28% specified whether sex was permitted with casual GBMSM partners. In its current form, the agreement builder activity enabled couples to choose and select items for their SA from a menu consisting of 5 categories with a total of 96 items. This approach, although deemed to be acceptable in our formative work leading to the pilot trial, may have diminished the focus on HIV/STI prevention and overwhelmed some of the partners/couples given the array of choices. It is also possible that some of the couples may have perceived their risk for HIV/STIs to be low and opted to not include any items about prevention. Prior research has found that couples perceived their risk for HIV and other STIs to be generally low, in part because of their beliefs that being in a relationship—by virtue—incorrectly reduces their risk or protects them from HIV/STIs [79]. One possible solution to encourage couples to include HIV/STI prevention items in their SA is to restructure, streamline, and simplify the agreement builder activity. First, an electronic algorithm could be embedded in the activity to prompt each partner of the couple to answer a brief set of questions to gauge the kind of sexual relationship they would want and the types of sexual behaviors they would prefer to engage in. Their responses to these questions could then automatically generate and place HIV/STI prevention items in their agreement for a more directed approach. Furthermore, the agreement builder activity could be broken down into several segments for couples to complete over time and not in one sitting. For example, once a couple decides which HIV/STI prevention items to include in their SA, they could then be prompted to revisit the agreement builder activity to focus on a different area that they deem to be important, such as strengthening and affirming their relationship. Changing the agreement builder activity is these ways (ie, algorithm, directed, and staggered) may help encourage couples to use the toolkit over time and simplify the process of building an agreement that meets their prevention and relationship needs (while lessening their feelings of being overwhelmed by too many choices).

### Sexual Agreement Outcomes

The preliminary impact of the eHealth HIV prevention toolkit intervention on couples’ establishment and adherence to an SA was also assessed. Compared with couples in the control arm, more couples in the intervention arm established an SA over time. Although a significant difference for establishing an SA was found at the 6-month follow-up between the 2 trial arms, the pilot trial was not adequately powered as we were more interested in obtaining point estimates and trends. These findings show initial promise for the toolkit intervention to help encourage couples who did not have an SA to establish one. However, there may be other possibilities that influenced couples to establish an SA, either apart (for couples in either trial arm) or in addition to using the toolkit (intervention arm only). Prior research has described that for some couples, certain circumstances or experiences (eg, events with others and influences from peers) may have led them to forming an SA [22]. It is also possible that couples established an SA as part of their natural progression in the relationship [19,31] and to enhance or improve an aspect of their relationship (eg, trust and intimacy) [21]. Future couples-based research that includes the establishment of an SA in the intervention would benefit to include an evaluation item to assess what influenced couples to form an agreement in their relationship.

A number of common relationship dynamics (eg, constructive communication, intimacy, and communal coping strategies to reduce HIV threat) at the averaged couple level were positively associated with couples establishing an SA—in general and over time. Similar findings were noted for lower averaged partner score differences being positively associated with couples establishing an SA. These findings align with what prior research with male couples has highlighted [6,26,80,81]: including and bolstering relationship dynamics along with sexual identity affirmation in couples-based interventions is critically important for HIV/STI prevention. It should be noted that findings from this trial suggest men’s perceptions about how much stigma there is for being gay in their local community and for being in a same-sex relationship may play an important role in HIV/STI prevention with male couples by decreasing their odds of establishing an SA. Specifically, as scores of the averaged couple level and differences between partners increase for these measures, the odds of a couple establishing an agreement decrease between 65% and 79%. Limited research has investigated the role that male couples’ living and social environment(s) may have toward their risk for HIV/STIs [80,82,83], particularly with respect to internalized and perceived stigma. Further research is warranted to examine the ways in which stigma may impact male couples’ relationships and efforts related to HIV/STI prevention.

With respect to adherence, fewer couples in the intervention arm broke their SA over time compared with couples in the control arm. Differences between the 2 trial arms were nonsignificant for both follow-up time points. Sample size constraints prevented our ability to quantitatively assess and meaningfully detect whether any differences in relationship dynamics existed between couples who broke their agreement compared with those who adhered to their agreement. A future trial with a larger sample size and longer follow-up time period (eg, 12 or 18 months) may provide a greater likelihood to assess any differences between couples who adhered to and did not adhere to their agreement, as had been found in a recent longitudinal study with male couples [84]. In addition, nonadherence to an SA may be defined differently between partners of the couple, which could influence how they might report about it. Recent research with male couples has found partner’s reports on what components and behaviors their agreement included did not always align [17,78], which could in turn affect their understanding of the agreement and their report of adherence. As such, better measurements are needed to improve detection of agreement breaks by considering the different components (eg, emotional and sexual) of a couples’ agreement.

### Limitations

This pilot RCT has several limitations. A convenience sample was recruited by placement of targeted advertisements on Facebook, thereby limiting the generalizability of the study’s findings as not all partnered men may use Facebook and those who do may not respond to advertisements about participating in HIV prevention or relationship research studies. Second, establishment and adherence to an SA were assessed by self-reporting. Social desirability bias may have influenced participants’ responses to these survey items, thereby potentially affecting the study’s outcome findings. The study also did not include serodiscordant and seroconcordant positive male couples or partnered transgender individuals (eg, transmen)—other populations who are in need of accessible, couples-based HIV/STI prevention interventions. A future iteration of the toolkit should include the biomedical (eg, Undetectable=Untransmissible and TasP), behavioral, and relational needs of serodiscordant and seroconcordant positive male couples [85] and transgender individuals and their relationship partners. Despite these limitations, findings from this pilot study showed promise for encouraging couples to establish and adhere to their SAs to warrant continuation of this research for HIV/STI prevention. A future trial of the updated toolkit with a larger sample size would provide sufficient power to detect effects and changes over time to assess whether establishing and adhering to an SA could enhance HIV/STI prevention efforts for male couples.

### Conclusions

Our findings demonstrate strong evidence for the acceptability and feasibility of the eHealth toolkit as a brief, stand-alone, couples-based HIV/STI prevention intervention. These findings support the need to update the toolkit and evaluate it in a larger clinical trial powered for efficacy. Moreover, this intervention could be combined and/or supplemented with other couples-based HIV/STI prevention interventions such as CHTC to emphasize the importance of improving couple’s relationship functioning—via agreements—for HIV/STI prevention. To date, most current and upcoming couples-based HIV/STI prevention interventions for male couples have focused on outcomes of HIV/STI testing, condom use, PrEP, and/or ART and less so on outcomes of SA formation and adherence. This intervention helps to fill this gap in couples-based HIV/STI prevention services for male couples.

## Appendix

Multimedia Appendix 1 Screener items with accompanying decision rules used for couple verification test.

Multimedia Appendix 2 Description of relationship dynamics assessed.

Multimedia Appendix 3 Couples' investment in a sexual agreement, by trial arm and assessment time point.

Multimedia Appendix 4 Results from multilevel logistic regression to predict the odds ratio of a couple establishing a sexual agreement over time for trial arm and relative to their averaged relationship dynamic score.

Multimedia Appendix 5 CONSORT-eHEALTH checklist (V 1.6.1).

## Abbreviations

ART: antiretroviral treatment
CAS: condomless anal sex
CHTC: couple’s HIV testing and counseling
CIT: couples interdependence theory
eHealth: electronic health
GBMSM: gay, bisexual, and other men who have sex with men
IP: Internet Protocol
LGBTQ: lesbian, gay, bisexual, transgender, and questioning/queer
mHealth: mobile health
OR: odds ratio
PrEP: pre-exposure prophylaxis
RCT: randomized controlled trial
SA: sexual agreement
SAIS: Sexual Agreement Investment Scale
STI: sexually transmitted infection
TasP: treatment as prevention
