# Supplementary material for: Feasibility and Acceptability of an Electronic Health HIV Prevention Toolkit Intervention With Concordant HIV-Negative, Same-Sex Male Couples on Sexual Agreement Outcomes: Pilot Randomized Controlled Trial
Source: JMIR Form Res. 2020 Feb 11;4(2):e16807. doi: 10.2196/16807 (PMC7058171; doi:10.2196/16807)
Supplement: Multimedia Appendix 1 [file formative_v4i2e16807_app1.doc]

**Multimedia Appendix 1.** Screener items with accompanying decision rules used

**for couple verification test**

| **Relationship verification rules for responses, by item between both partners of the couple** | | |
| --- | --- | --- |
| **Item** | **Reported by Partner 1 (index)** | **Reported by Partner 2** |
| 1. Partner 1 age | —^a^ | +/- 1 year |
| 1. Partner 2 age | +/- 1 year | —^a^ |
| 2. Partner 1 birthday month | —^a^ | Exact |
| 2. Partner 2 birthday month | Exact | —^a^ |
| 3. Relationship length | Same response | Same response |
| 4. Establishment of a sexual agreement | Either disagreed or concurred about not having an agreement | Either disagreed or concurred about not having an agreement |
| 5. Partner 1 email / cell number | —^a^ | Must match one |
| 5. Partner 2 email / cell number | Must match one | —^a^ |

***Note***

^a^ Represents self-reports of one’s own information
