# Supplementary material for: Feasibility and Acceptability of an Electronic Health HIV Prevention Toolkit Intervention With Concordant HIV-Negative, Same-Sex Male Couples on Sexual Agreement Outcomes: Pilot Randomized Controlled Trial
Source: JMIR Form Res. 2020 Feb 11;4(2):e16807. doi: 10.2196/16807 (PMC7058171; doi:10.2196/16807)
Supplement: Multimedia Appendix 2 [file formative_v4i2e16807_app2.doc]

**Multimedia Appendix 2. Description of relationship dynamics assessed**

| **Scale** | **Brief description** | **# items** | **Likert-type response range** | **Cronbach’s alpha** |
| --- | --- | --- | --- | --- |
| Dyadic trust [67] | Captures perceptions of interpersonal trust in close relationships (e.g., couples). | 8 | 1 (Strongly disagree) to 5 (Strongly agree). | 0.91 |
| Investment Model for Relationship Commitment - Shortened [68] | Assesses participants’ commitment to being in the relationship with their current partner. Items addressed commitment, satisfaction, investment size, and quality of alternatives. | 13 | 0 (Do not agree at all) to 6 (Agree completely). | 0.87 |
| Relationship satisfaction [69] | Measures overall relationship satisfaction. | 7 | 1 (Low) to 5 (High satisfaction) | 0.89 |
| Miller Social Intimacy [71] | Assesses intimacy in the context of close friendship / relationship / marriage, with items on frequency and intensity of perceived intimacy with the other person. Higher scores indicate greater amounts of social intimacy. | 17 | 1 (Very rarely/ not much) to 10 (Always). | 0.90 |
| Communication Patterns Questionnaire – Short Form [72] | Self-assessment of partners perceptions of relational interactions of when an issue or problem arises and during related discussions relative to avoidance and withdrawal (8 items, 0.77) and constructive behaviors (3 items, 0.82). | 11 | 1 (Very unlikely) to 9 (Very likely) | 0.82 |
| Communal confidence [73] | Assesses participants level of agreement with their partner on broad topics related to their relationship: 1) Life philosophy; 2) Aims, goals, and things believed important; 3) Amount of time spent together; 4) Having a stimulating exchange of ideas; 5) Calmly discussing something together; 6) Working together on a project | 6 | 1 (Always disagree), to 6 (Always agree) | 0.84 |
| **Scale** | **Brief description** | **# items** | **Likert-type response range** | **Cronbach’s alpha** |
| Sexual agreement investment scale [75] ^a^ | Assesses the level of investment in a sexual agreement by considering one’s commitment to, satisfaction in, and value toward the agreement they have with their partner. | 13 | 0 (Not at all) to 4 (Extremely) | 0.95 |
| Use of communal coping strategies to reduce HIV threat [73] | Assesses how often partners engage in joint efforts to manage the health threat of HIV infection via items on 1) couple communication about  behavior change of different prevention strategies, 2) working together for different prevention strategies, and 3) making decisions together about using different prevention strategies. The 7 prevention strategies are: (a) using condoms when having sex with each other; (b) limiting the number of other sex partners; (c) either of us having sex ‘outside’ our relationship; (d) using condoms when either of us has sex outside our relationship; (e) getting tested regularly for STIs and HIV; (f) being the top or bottom when we have sex with each other; (g) being sexually faithful to each other. | 21 | 1 (Not to any extent at all) to 5 (To a great extent) | 0.95 |
|  |  |  |  |  |
| Preferences for general lifestyle outcomes [73] | Assessed the degree to which interacting partners agree about the shared or joint outcomes in their relationship on (a) what we like to eat, (b) whether to exercise, (c) money matters, (d) preventive health (e.g., flu shots, general check-up, and dental), (e) our social activities, and (f) our sexual relationship. | 6 | 1 (We don’t agree at all) to 5 (We agree completely) | 0.63 |
| **Scale** | **Brief description** | **# items** | **Likert-type response range** | **Cronbach’s alpha** |
| Preferences for sexual health outcomes [73] | Assesses the extent the participant and his male partner were concordant when it came to sexual health topics of: (a) using condoms when having sex  with each other; (b) limiting the number of other sex partners; (c) either of us having sex ‘outside’ our relationship; (d) using condoms when either of us has sex outside our relationship; (e) getting tested regularly for STIs and HIV; (f) being the top or bottom when we have sex with each other; (g) being sexually faithful to each other. | 7 | 1 (We don’t agree at all) to 5 (We agree completely) | 0.82 |
| HIV social support scale [74] | Assesses the level of HIV specific social support from one’s partner or specifically support received and given around managing HIV risk. | 24 | 1 (Strongly disagree) to 4 (Strongly agree) | 0.84 |
| HIV-negative couples’ perceptions of severity of HIV infection [73] | Assesses the perception of the personal, psychosocial, and physical consequences of a particular health threat: HIV infection. | 13 | 1 (Strongly disagree/Not at all serious) to 5 (Strongly agree / Very serious) | 0.89 |
| Perceptions of local stigma [76] | Assesses one’s perceptions of stigma related to being gay in their current community. | 7 | 1 (Strongly disagree) to 5 (Strongly agree) | 0.89 |
| Perceptions of gay-related stigma [73] | Assesses participants’ personal perceptions of how much stigma there is for being in a gay, same-sex relationship (i.e., gay couple). | 6 | 1 (None at all) to 5 (A lot). | 0.73 |
| Internalized homophobia [77] | Developed for use with gay men to measure the extent to which negative attitudes and beliefs about homosexuality are internalized and integrated into one's self-image and gay identity. | 12 | 1 (Strongly disagree) to 5 (Strongly agree) | 0.81 |

***Note***

^a^ Only asked at 3- and 6-month assessments
