# Supplementary material for: Feasibility and Acceptability of an Electronic Health HIV Prevention Toolkit Intervention With Concordant HIV-Negative, Same-Sex Male Couples on Sexual Agreement Outcomes: Pilot Randomized Controlled Trial
Source: JMIR Form Res. 2020 Feb 11;4(2):e16807. doi: 10.2196/16807 (PMC7058171; doi:10.2196/16807)
Supplement: Multimedia Appendix 3 [file formative_v4i2e16807_app3.doc]

**Multimedia Appendix 3.** Couples’ investment in a sexual agreement, by trial arm and assessment time point

| **3-month assessment** | **Cohort, 64 couples ^a^** | **Intervention, 29 couples** | **Control, 35 couples** | **Effect size (*d*)** | ***P value*** |
| --- | --- | --- | --- | --- | --- |
| **Sexual agreement investment scale, mean (SD)** | | | | | |
| Partner score | 3.56 (0.60) | 3.58 (0.56) | 3.54 (0.63) | 0.067 | 0.65 |
| Score difference between  partners | 0.59 (0.59) | 0.54 (0.56) | 0.63 (0.63) | 0.150 | 0.55 |
| **Commitment, mean (SD)** | | | | | |
| Partner score | 3.66 (0.59) | 3.68 (0.51) | 3.65 (0.65) | 0.051 | 0.69 |
| Score difference between  partners | 0.52 (0.67) | 0.45 (0.57) | 0.59 (0.74) | 0.209 | 0.42 |
| **Satisfaction, mean (SD)** | | | | | |
| Partner score | 3.45 (0.74) | 3.46 (0.70) | 3.45 (0.78) | 0.014 | 0.91 |
| Score difference between  partners | 0.71 (0.68) | 0.69 (0.73) | 0.72 (0.65) | 0.044 | 0.66 |
| **Value, mean (SD)** | | | | | |
| Partner score | 3.55 (0.63) | 3.58 (0.63) | 3.52 (0.64) | 0.095 | 0.53 |
| Score difference between  partners | 0.59 (0.64) | 0.56 (0.62) | 0.62 (0.67) | 0.093 | 0.71 |
| **6-month assessment** | **Cohort, 59 couples ^a^** | **Intervention, 32 couples** | **Control, 27 couples** | **Effect size (*d*)** | ***P value*** |
| **Sexual agreement investment scale, mean (SD)** | | | | | |
| Partner score | 3.55 (0.64) | 3.55 (0.61) | 3.56 (0.66) | 0.016 | 0.94 |
| Score difference between  partners | 0.57 (0.70) | 0.49 (0.71) | 0.66 (0.69) | 0.243 | 0.35 |
| **Commitment, mean (SD)** | | | | | |
| Partner score | 3.66 (0.59) | 3.65 (0.60) | 3.67 (0.59) | 0.034 | 0.77 |
| Score difference between  partners | 0.46 (0.66) | 0.42 (0.73) | 0.50 (0.57) | 0.122 | 0.65 |
| **Satisfaction, mean (SD)** | | | | | |
| Partner score | 3.42 (0.79) | 3.43 (0.71) | 3.42 (0.86) | 0.013 | 0.98 |
| Score difference between  partners | 0.68 (0.83) | 0.54 (0.70) | 0.84 (0.95) | 0.354 | 0.17 |
| **Value, mean (SD)** | | | | | |
| Partner score | 3.55 (0.65) | 3.55 (0.64) | 3.55 (0.67) | 0 | 0.91 |
| Score difference between  partners | 0.61 (0.74) | 0.55 (0.74) | 0.69 (0.75) | 0.188 | 0.50 |

***Note***

^a^ Sample size represents couples who concurred about establishing a sexual agreement
