# Supplementary material for: Feasibility and Acceptability of an Electronic Health HIV Prevention Toolkit Intervention With Concordant HIV-Negative, Same-Sex Male Couples on Sexual Agreement Outcomes: Pilot Randomized Controlled Trial
Source: JMIR Form Res. 2020 Feb 11;4(2):e16807. doi: 10.2196/16807 (PMC7058171; doi:10.2196/16807)
Supplement: Multimedia Appendix 4 [file formative_v4i2e16807_app4.doc]

**Multimedia Appendix 4.** Results from multilevel logistic regression to predict the odds ratio of a couple establishing a sexual agreement over time for trial arm and relative to their averaged relationship dynamic score.

| Couple averaged variable and covariates | | Multilevel logistic regression | | Couple-averaged variable by establishment of sexual agreement | | |
| --- | --- | --- | --- | --- | --- | --- |
|  |  | Odds ratio (95% CI) | *P* value | Yes, mean (SD) | No, mean (SD) | Effect size (d) |
|  | |  |  |  |  |  |
| **Dyadic trust** | | 2.01 (0.94-4.32) | .07 | 4.27 (0.56) | 4.12 (0.60) | 0.26 |
|  | Assessment at 6 months versus 3 months | 0.95 (0.53-1.71) | .87 | — | — | — |
|  | Intervention versus control | 2.12 (0.78-5.70) | .14 | — | — | — |
| **Relationship commitment—partner difference** | | 0.92 (0.49-1.71) | .79 | 0.75 (0.67) | 0.76 (0.67) | 0.02 |
|  | Assessment at 6 months versus 3 months | 0.93 (0.52-1.68) | .82 | — | — | — |
|  | Intervention versus control | 2.24 (0.84-5.97) | .10 | — | — | — |
| **Relationship satisfaction** | | 3.08 (1.45-6.55) | <.01 | 4.45 (0.58) | 4.24 (0.70) | 0.32 |
|  | Assessment at 6 months versus 3 months | 0.93 (0.52-1.66) | .80 | — | — | — |
|  | Intervention versus control | 1.84 (0.62-5.42) | .27 | — | — | — |
| **Sexual satisfaction with relationship—partner difference** | | 0.95 (0.50-1.81) | .89 | 0.76 (0.64) | 0.76 (0.57) | 0 |
|  | Assessment at 6 months versus 3 months | 0.93 (0.52-1.66) | .80 | — | — | — |
|  | Intervention versus control | 2.31 (0.86-6.16) | .09 | — | — | — |
| **Social intimacy** | | 1.88 (1.07-3.32) | .03 | 8.72 (0.72) | 8.42 (0.87) | 0.37 |
|  | Assessment at 6 months versus 3 months | 0.96 (0.53-1.71) | .88 | — | — | — |
|  | Intervention versus control | 2.04 (0.75-5.56) | .17 | — | — | — |
| **Social intimacy difference** | | 0.84 (0.52-1.37) | .49 | 0.80 (0.76) | 0.95 (0.83) | 0.19 |
|  | Assessment at 6 months versus 3 months | 0.94 (0.53-1.68) | .84 | — | — | — |
|  | Intervention versus control | 2.30 (0.87-6.10) | .09 | — | — | — |
| **Avoidance and withdrawal communication—partner difference** | | 0.86 (0.52-1.41) | .54 | 1.17 (1.05) | 1.24 (0.95) | 0.07 |
|  | Assessment at 6 months versus 3 months | 0.92 (0.51-1.66) | .78 | — | — | — |
|  | Intervention versus control | 2.34 (0.84-6.50) | .10 | — | — | — |
| **Constructive communication** | | 1.46 (1.08-1.96) | .01 | 7.03 (1.36) | 6.45 (1.54) | 0.40 |
|  | Assessment at 6 months versus 3 months | 0.87 (0.49-1.57) | .65 | — | — | — |
|  | Intervention versus control | 2.33 (0.86-6.31) | .09 | — | — | — |
| **Constructive communication—partner difference** | | 0.93 (0.68-1.27) | .66 | 1.45 (1.36) | 1.49 (1.09) | 0.03 |
|  | Assessment at 6 months versus 3 months | 0.91 (0.51-1.63) | .74 | — | — | — |
|  | Intervention versus control | 2.30 (0.86-6.16) | .10 | — | — | — |
| **Communal confidence** | | 1.13 (1.01-1.26) | .03 | 29.06 (3.69) | 27.52 (4.05) | 0.40 |
|  | Assessment at 6 months versus 3 months | 0.85 (0.47-1.52) | .57 | — | — | — |
|  | Intervention versus control | 1.94 (0.73-5.12) | .18 | — | — | — |
| **Communal coping strategies to reduce HIV threat** | | 4.22 (2.04-8.73) | <.01 | 4.30 (0.50) | 3.89 (0.65) | 0.70 |
|  | Assessment at 6 months versus 3 months | 0.76 (0.41-1.38) | .36 | — | — | — |
|  | Intervention versus control | 1.91 (0.71-5.11) | .20 | — | — | — |
| **Communal coping strategies to reduce HIV threat—partner difference** | | 0.94 (0.56-1.57) | .81 | 0.81 (0.76) | 0.88 (0.77) | 0.09 |
|  | Assessment at 6 months versus 3 months | 0.93 (0.52-1.65) | .79 | — | — | — |
|  | Intervention versus control | 2.26 (0.84-6.10) | .10 | — | — | — |
| **Preferences for sexual health outcomes** | | 1.05 (0.90-1.22) | .54 | 32.13 (2.90) | 31.59 (3.14) | 0.18 |
|  | Assessment at 6 months versus 3 months | 0.93 (0.52-1.67) | .81 | — | — | — |
|  | Intervention versus control | 2.23 (0.85-5.89) | .10 | — | — | — |
| **Preferences for sexual health outcomes—partner difference** | | 0.93 (0.82-1.05) | .23 | 3.30 (3.36) | 3.93 (3.87) | 0.02 |
|  | Assessment at 6 months versus 3 months | 0.89 (0.50-1.60) | .70 | — | — | — |
|  | Intervention versus control | 2.34 (0.88-6.24) | .09 | — | — | — |
| **HIV social support—partner difference** | | 0.54 (0.14-2.15) | .38 | 0.37 (0.28) | 0.37 (0.31) | 0 |
|  | Assessment at 6 months versus 3 months | 0.89 (0.50-1.60) | .69 | — | — | — |
|  | Intervention versus control | 2.31 (0.85-6.26) | .09 | — | — | — |
| **Perceptions of severity of HIV infection** | | 1.91 (0.95-3.83) | .07 | 3.77 (0.66) | 3.59 (0.64) | 0.28 |
|  | Assessment at 6 months versus 3 months | 1.01 (0.56-1.81) | .99 | — | — | — |
|  | Intervention versus control | 2.04 (0.74-5.64) | .17 | — | — | — |
| **Perceived local stigma** | | 0.21 (0.11-0.43) | <.01 | 3.73 (0.81) | 4.23 (0.63) | 0.70 |
|  | Assessment at 6 months versus 3 months | 0.97 (0.53-1.75) | .91 | — | — | — |
|  | Intervention versus control | 1.94 (0.64-5.92) | .24 | — | — | — |
| **Perceived local stigma—partner** | | 1.30 (0.75-2.27) | .36 | 0.80 (0.76) | 0.73 (0.69) | 0.10 |
|  | Assessment at 6 months versus 3 months | 0.93 (0.52-1.66) | .81 | — | — | — |
|  | Intervention versus control | 2.40 (0.89-6.47) | .08 | — | — | — |
| **Perceived gay-related stigma** | | 0.35 (0.16-0.79) | .01 | 4.06 (0.62) | 4.32 (0.50) | 0.47 |
|  | Assessment at 6 months versus 3 months | 0.95 (0.53-1.69) | .85 | — | — | — |
|  | Intervention versus control | 2.53 (0.95-6.75) | .06 | — | — | — |
| **Perceived gay-related stigma—partner difference** | | 1.33 (0.59-3.02) | .49 | 0.59 (0.49) | 0.59 (0.50) | 0 |
|  | Assessment at 6 months versus 3 months | 0.95 (0.53-1.71) | .87 | — | — | — |
|  | Intervention versus control | 2.38 (0.88-6.43) | .09 | — | — | — |
| **Internalized homophobia** | | 1.29 (0.52-3.17) | .58 | 1.70 (0.51) | 1.66 (0.47) | 0.08 |
|  | Assessment at 6 months versus 3 months | 0.93 (0.52-1.66) | .80 | — | — | — |
|  | Intervention versus control | 2.26 (0.84-6.10) | .10 | — | — | — |
| **Internalized homophobia—partner difference** | | 0.77 (0.37-1.61) | .49 | 0.54 (0.51) | 0.62 (0.61) | 0.14 |
|  | Assessment at 6 months versus 3 months | 0.92 (0.51-1.64) | .77 | — | — | — |
|  | Intervention versus control | 2.33 (0.88-6.20) | .09 | — | — | — |
