# Supplementary material for: Feasibility and Acceptability of an Electronic Health HIV Prevention Toolkit Intervention With Concordant HIV-Negative, Same-Sex Male Couples on Sexual Agreement Outcomes: Pilot Randomized Controlled Trial
Source: JMIR Form Res. 2020 Feb 11;4(2):e16807. doi: 10.2196/16807 (PMC7058171; doi:10.2196/16807)
Supplement: Multimedia Appendix 5 [file formative_v4i2e16807_app5.pdf]

CONSORT-EHEALTH (V 1.6.1) - Submission/Publication Form

The CONSORT-EHEALTH checklist is intended for authors of randomized trials evaluating web-based and Internet-based applications/interventions, including mobile interventions, electronic games (incl multiplayer games), social media, certain telehealth applications, and other interactive and/or networked electronic applications. Some of the items (e.g. all subitems under item 5 - description of the intervention) may also be applicable for other study designs.

The goal of the CONSORT EHEALTH checklist and guideline is to be
a) a guide for reporting for authors of RCTs,
b) to form a basis for appraisal of an ehealth trial (in terms of validity)

CONSORT-EHEALTH items/subitems are MANDATORY reporting items for studies published in the Journal of Medical Internet Research and other journals / scientific societies endorsing the checklist.

Items numbered 1., 2., 3., 4a., 4b etc are original CONSORT or CONSORT-NPT (non-pharmacologic treatment) items.
Items with Roman numerals (i., ii, iii, iv etc.) are CONSORT-EHEALTH extensions/clarifications.

As the CONSORT-EHEALTH checklist is still considered in a formative stage, we would ask that you also RATE ON A SCALE OF 1-5 how important/useful you feel each item is FOR THE PURPOSE OF THE CHECKLIST and reporting guideline (optional).

Mandatory reporting items are marked with a red *.
In the textboxes, either copy & paste the relevant sections from your manuscript into this form - please include any quotes from your manuscript in QUOTATION MARKS,
or answer directly by providing additional information not in the manuscript, or elaborating on why the item was not relevant for this study.

YOUR ANSWERS WILL BE PUBLISHED AS A SUPPLEMENTARY FILE TO YOUR PUBLICATION IN JMIR AND ARE CONSIDERED PART OF YOUR PUBLICATION (IF ACCEPTED).
Please fill in these questions diligently. Information will not be copyedited, so please use proper spelling and grammar, use correct capitalization, and avoid abbreviations.

DO NOT FORGET TO SAVE AS PDF _AND_ CLICK THE SUBMIT BUTTON SO YOUR ANSWERS ARE IN OUR DATABASE !!!

Citation Suggestion (if you append the pdf as Appendix we suggest to cite this paper in the caption):
Eysenbach G, CONSORT-EHEALTH Group
CONSORT-EHEALTH: Improving and Standardizing Evaluation Reports of Web-based and

Mobile Health Interventions
J Med Internet Res 2011;13(4):e126
URL: http://www.jmir.org/2011/4/e126/
doi: 10.2196/jmir.1923
PMID: 22209829

* Required

Your name *

First Last

Jason W. Mitchell

Primary Affiliation (short), City, Country *

University of Toronto, Toronto, Canada

University of Hawaii, Honolulu, USA

Your e-mail address *

abc@gmail.com

jasonmit@hawaii.edu

Title of your manuscript *

Provide the (draft) title of your manuscript.

Feasibility and Acceptability of an Electronic Health HIV Prevention Toolkit Intervention With Concordant HIV-Negative, Same-Sex Male Couples on Sexual Agreement Outcomes: Pilot Randomized Controlled Trial

Name of your App/Software/Intervention *

If there is a short and a long/alternate name, write the short name first and add the long name in brackets.

Electronic Health HIV Prevention Toolkit Intern

Evaluated Version (if any)

e.g. "V1", "Release 2017-03-01", "Version 2.0.27913"

Your answer

Language(s) *

What language is the intervention/app in? If multiple languages are available, separate by comma (e.g. "English, French")

English

URL of your Intervention Website or App

e.g. a direct link to the mobile app on app in appstore (itunes, Google Play), or URL of the website. If the intervention is a DVD or hardware, you can also link to an Amazon page.

Your answer

URL of an image/screenshot (optional)

Your answer

Accessibility *

Can an enduser access the intervention presently?

- ◯ access is free and open
- ◯ access only for special usergroups, not open
- ◯ access is open to everyone, but requires payment/subscription/in-app purchases
- ◉ app/intervention no longer accessible
- ◯ Other:

Primary Medical Indication/Disease/Condition *

e.g. "Stress", "Diabetes", or define the target group in brackets after the condition, e.g. "Autism (Parents of children with)", "Alzheimers (Informal Caregivers of)"

HIV prevention (sero-concordant negative mal[illegible]

Primary Outcomes measured in trial *

comma-separated list of primary outcomes reported in the trial

establishment of a sexual agreement, adheren

Secondary/other outcomes

Are there any other outcomes the intervention is expected to affect?

Improvement in relationship dynamics / functioning

Recommended "Dose" *

What do the instructions for users say on how often the app should be used?

- ( ) Approximately Daily
- ( ) Approximately Weekly
- ( ) Approximately Monthly
- ( ) Approximately Yearly
- ( ) "as needed"
- (•) Other: As often as men and couples want to use it

Approx. Percentage of Users (starters) still using the app as recommended after 3 months *

- (•) unknown / not evaluated
- ( ) 0-10%
- ( ) 11-20%
- ( ) 21-30%
- ( ) 31-40%
- ( ) 41-50%
- ( ) 51-60%
- ( ) 61-70%
- ( ) 71%-80%
- ( ) 81-90%
- ( ) 91-100%
- ( ) Other:

Overall, was the app/intervention effective? *

- ( ) yes: all primary outcomes were significantly better in intervention group vs control
- (x) partly: SOME primary outcomes were significantly better in intervention group vs control
- ( ) no statistically significant difference between control and intervention
- ( ) potentially harmful: control was significantly better than intervention in one or more outcomes
- ( ) inconclusive: more research is needed
- ( ) Other:

Article Preparation Status/Stage *

At which stage in your article preparation are you currently (at the time you fill in this form)

- ( ) not submitted yet - in early draft status
- ( ) not submitted yet - in late draft status, just before submission
- ( ) submitted to a journal but not reviewed yet
- ( ) submitted to a journal and after receiving initial reviewer comments
- (x) submitted to a journal and accepted, but not published yet
- ( ) published
- ( ) Other:

Journal *

If you already know where you will submit this paper (or if it is already submitted), please provide the journal name (if it is not JMIR, provide the journal name under "other")

- ○ not submitted yet / unclear where I will submit this
- ○ Journal of Medical Internet Research (JMIR)
- ○ JMIR mHealth and UHealth
- ○ JMIR Serious Games
- ○ JMIR Mental Health
- ○ JMIR Public Health
- ◉ JMIR Formative Research
- ○ Other JMIR sister journal
- ○ Other:

Is this a full powered effectiveness trial or a pilot/feasibility trial? *

- ◉ Pilot/feasibility
- ○ Fully powered

Manuscript tracking number *

If this is a JMIR submission, please provide the manuscript tracking number under "other" (The ms tracking number can be found in the submission acknowledgement email, or when you login as author in JMIR. If the paper is already published in JMIR, then the ms tracking number is the four-digit number at the end of the DOI, to be found at the bottom of each published article in JMIR)

- ○ no ms number (yet) / not (yet) submitted to / published in JMIR
- ◉ Other: 16807

TITLE AND ABSTRACT

1a) TITLE: Identification as a randomized trial in the title

1a) Does your paper address CONSORT item 1a? *

I.e does the title contain the phrase "Randomized Controlled Trial"? (if not, explain the reason under "other")

- (●) yes
- ( ) Other:

1a-i) Identify the mode of delivery in the title

Identify the mode of delivery. Preferably use “web-based” and/or “mobile” and/or “electronic game” in the title. Avoid ambiguous terms like “online”, “virtual”, “interactive”. Use “Internet-based” only if Intervention includes non-web-based Internet components (e.g. email), use “computer-based” or “electronic” only if offline products are used. Use “virtual” only in the context of “virtual reality” (3-D worlds). Use “online” only in the context of “online support groups”. Complement or substitute product names with broader terms for the class of products (such as “mobile” or “smart phone” instead of “iphone”), especially if the application runs on different platforms.

	1	2	3	4	5	
subitem not at all important	( )	( )	( )	( )	(●)	essential

Does your paper address subitem 1a-i? *

Copy and paste relevant sections from manuscript title (include quotes in quotation marks "like this" to indicate direct quotes from your manuscript), or elaborate on this item by providing additional information not in the ms, or briefly explain why the item is not applicable/relevant for your study

"Electronic Health"

1a-ii) Non-web-based components or important co-interventions in title

Mention non-web-based components or important co-interventions in title, if any (e.g., “with telephone support”).

	1	2	3	4	5	
subitem not at all important	(●)	( )	( )	( )	( )	essential

Does your paper address subitem 1a-ii?

Copy and paste relevant sections from manuscript title (include quotes in quotation marks "like this" to indicate direct quotes from your manuscript), or elaborate on this item by providing additional information not in the ms, or briefly explain why the item is not applicable/relevant for your study

all components in intervention were in the interactive website ("Electronic Health HIV Prevention Toolkit Intervention")

1a-iii) Primary condition or target group in the title

Mention primary condition or target group in the title, if any (e.g., “for children with Type I Diabetes”) Example: A Web-based and Mobile Intervention with Telephone Support for Children with Type I Diabetes: Randomized Controlled Trial

	1	2	3	4	5	
subitem not at all important	( )	( )	( )	( )	(●)	essential

Does your paper address subitem 1a-iii? *

Copy and paste relevant sections from manuscript title (include quotes in quotation marks "like this" to indicate direct quotes from your manuscript), or elaborate on this item by providing additional information not in the ms, or briefly explain why the item is not applicable/relevant for your study

Yes: "HIV-negative, same-sex male couples"

1b) ABSTRACT: Structured summary of trial design, methods, results, and conclusions

NPT extension: Description of experimental treatment, comparator, care providers, centers, and blinding status.

1b-i) Key features/functionalities/components of the intervention and comparator in the METHODS section of the ABSTRACT

Mention key features/functionalities/components of the intervention and comparator in the abstract. If possible, also mention theories and principles used for designing the site. Keep in mind the needs of systematic reviewers and indexers by including important synonyms. (Note: Only report in the abstract what the main paper is reporting. If this information is missing from the main body of text, consider adding it)

	1	2	3	4	5	
subitem not at all important	○	○	◉	○	○	essential

Does your paper address subitem 1b-i? *

Copy and paste relevant sections from the manuscript abstract (include quotes in quotation marks "like this" to indicate direct quotes from your manuscript), or elaborate on this item by providing additional information not in the ms, or briefly explain why the item is not applicable/relevant for your study

Yes, but not in abstract. This information is provided in Methods section and subsection Description of the Electronic Health HIV Prevention Toolkit Intervention

1b-ii) Level of human involvement in the METHODS section of the ABSTRACT

Clarify the level of human involvement in the abstract, e.g., use phrases like “fully automated” vs. “therapist/nurse/care provider/physician-assisted” (mention number and expertise of providers involved, if any). (Note: Only report in the abstract what the main paper is reporting. If this information is missing from the main body of text, consider adding it)

	1	2	3	4	5	
subitem not at all important	○	○	◉	○	○	essential

Does your paper address subitem 1b-ii?

Copy and paste relevant sections from the manuscript abstract (include quotes in quotation marks "like this" to indicate direct quotes from your manuscript), or elaborate on this item by providing additional information not in the ms, or briefly explain why the item is not applicable/relevant for your study

Yes, but not in abstract. This information is provided in Methods section and subsection Description of the Electronic Health HIV Prevention Toolkit Intervention

1b-iii) Open vs. closed, web-based (self-assessment) vs. face-to-face assessments in the METHODS section of the ABSTRACT

Mention how participants were recruited (online vs. offline), e.g., from an open access website or from a clinic or a closed online user group (closed usergroup trial), and clarify if this was a purely web-based trial, or there were face-to-face components (as part of the intervention or for assessment). Clearly say if outcomes were self-assessed through questionnaires (as common in web-based trials). Note: In traditional offline trials, an open trial (open-label trial) is a type of clinical trial in which both the researchers and participants know which treatment is being administered. To avoid confusion, use “blinded” or “unblinded” to indicated the level of blinding instead of “open”, as “open” in web-based trials usually refers to “open access” (i.e. participants can self-enrol). (Note: Only report in the abstract what the main paper is reporting. If this information is missing from the main body of text, consider adding it)

	1	2	3	4	5	
subitem not at all important	○	○	○	○	◉	essential

Does your paper address subitem 1b-iii?

Copy and paste relevant sections from the manuscript abstract (include quotes in quotation marks "like this" to indicate direct quotes from your manuscript), or elaborate on this item by providing additional information not in the ms, or briefly explain why the item is not applicable/relevant for your study

Yes, but not in abstract. This information is provided in the Methods section of the manuscript.

1b-iv) RESULTS section in abstract must contain use data

Report number of participants enrolled/assessed in each group, the use/uptake of the intervention (e.g., attrition/adherence metrics, use over time, number of logins etc.), in addition to primary/secondary outcomes. (Note: Only report in the abstract what the main paper is reporting. If this information is missing from the main body of text, consider adding it)

	1	2	3	4	5	
subitem not at all important	○	○	○	○	◉	essential

Does your paper address subitem 1b-iv?

Copy and paste relevant sections from the manuscript abstract (include quotes in quotation marks "like this" to indicate direct quotes from your manuscript), or elaborate on this item by providing additional information not in the ms, or briefly explain why the item is not applicable/relevant for your study

Yes, like this "Overall, 7959 individuals initiated screening. Reasons for individual ineligibility varied. An electronic algorithm was used to assess couple-level eligibility, which identified 1080 ineligible and 266 eligible dyads. 149 eligible couples enrolled into the pilot RCT: 68 received the intervention and 81 received the education control. Retention was 71.5% (213/298 partnered men) over the 6 months. Participants reported high acceptability of the intervention along with some areas for improvement." Much more information is provided in the Results section of the manuscript.

1b-v) CONCLUSIONS/DISCUSSION in abstract for negative trials

Conclusions/Discussions in abstract for negative trials: Discuss the primary outcome - if the trial is negative (primary outcome not changed), and the intervention was not used, discuss whether negative results are attributable to lack of uptake and discuss reasons. (Note: Only report in the abstract what the main paper is reporting. If this information is missing from the main body of text, consider adding it)

	1	2	3	4	5	
subitem not at all important	○	○	○	◉	○	essential

Does your paper address subitem 1b-v?

Copy and paste relevant sections from the manuscript abstract (include quotes in quotation marks "like this" to indicate direct quotes from your manuscript), or elaborate on this item by providing additional information not in the ms, or briefly explain why the item is not applicable/relevant for your study

Yes, like this "The findings demonstrate strong evidence for the acceptability and feasibility of the eHealth toolkit as a brief, stand-alone, couples-based HIV prevention intervention." Much more information is provided in the Discussion section of the manuscript.

INTRODUCTION

2a) In INTRODUCTION: Scientific background and explanation of rationale

2a-i) Problem and the type of system/solution

Describe the problem and the type of system/solution that is object of the study: intended as stand-alone intervention vs. incorporated in broader health care program? Intended for a particular patient population? Goals of the intervention, e.g., being more cost-effective to other interventions, replace or complement other solutions? (Note: Details about the intervention are provided in “Methods” under 5)

	1	2	3	4	5	
subitem not at all important	○	○	○	○	◉	essential

Does your paper address subitem 2a-i? *

Copy and paste relevant sections from the manuscript (include quotes in quotation marks "like this" to indicate direct quotes from your manuscript), or elaborate on this item by providing additional information not in the ms, or briefly explain why the item is not applicable/relevant for your study

Yes, like this "National estimates indicate that between one-third and two-thirds of HIV infections among gay, bisexual, and other men who have sex with men (GBMSM) occur within primary relationships (ie, male couples) [1,2]. In response to these estimates, a growing interest in couples-based approaches to HIV prevention [3-10] has emerged to investigate how relationship dynamics may affect male couples' risk for HIV and other sexually transmitted infections (STIs) and the development of interventions for this population. There is a need to develop innovative dyadic interventions that provide couples with the behavioral skills to manage the risk of HIV transmission within their relationship."

2a-ii) Scientific background, rationale: What is known about the (type of) system

Scientific background, rationale: What is known about the (type of) system that is the object of the study (be sure to discuss the use of similar systems for other conditions/diagnoses, if appropiate), motivation for the study, i.e. what are the reasons for and what is the context for this specific study, from which stakeholder viewpoint is the study performed, potential impact of findings [2]. Briefly justify the choice of the comparator.

	1	2	3	4	5	
subitem not at all important	○	○	○	○	◉	essential

Does your paper address subitem 2a-ii? *

Copy and paste relevant sections from the manuscript (include quotes in quotation marks "like this" to indicate direct quotes from your manuscript), or elaborate on this item by providing additional information not in the ms, or briefly explain why the item is not applicable/relevant for your study

Yes, like this "Sexual agreements (SAs) are one dynamic of male couples' relationships that have implications for HIV/STI prevention, as supported by a number of investigations identified in a recent scoping view [17]. An SA is formed when partners have explicit conversations with decision making that leads them to having a mutual understanding about which sexual and other relational behaviors, they want to occur with each other (ie, in their relationship) and if applicable, with anyone else (eg, casual sex partners) [18,19]. To date, much research has been conducted about male couples' SAs, including circumstances and reasons for forming an agreement [18,20-23], investment in one [24-27], and adherence rates and disclosure, and reasons when an agreement is broken [17,18,28]. SAs are common among male couples [18,21,25,26,20,31], vary by type [18,19,31,32] and are dynamic, such that changes in composition or type may occur over time [33]. Types of SAs and the composition of these types come in many forms, yet they generally fall into 3 broad categories of closed, open with guidelines, and open without guidelines. A closed agreement represents that sex only occurs between the primary relationship partners, whereas an open agreement with or without guidelines permits certain (or any) sexual and relational behaviors to occur with casual sex partners.

Implicitly, SAs have direct implications for HIV/STI prevention as this dynamic pertains to couples' sexual behaviors, which may or may not affect their risk for HIV and other STIs. A recent scoping review summarized the associations reported from previous studies on male couples' establishment and adherence to the agreement and their engagement in condomless anal sex (CAS) within and/or outside of the relationship [17]. In general, negative associations were found between engagement in CAS outside of the relationship and couples who concurred about having an agreement (including type) and to adhering to it [17]. Other work has found that partnered GBMSM's likelihood of having had CAS within and outside of the relationship significantly decreased as their scores of being invested in the SA increased [34].

The associations between couples' SAs and their attitudes toward couple's HIV testing and counseling (CHTC) and pre-exposure prophylaxis (PrEP) [35] has also been explored [36-40]. Findings from these studies point for the need to tailor content and messaging that account for couples' perceived concerns and benefits about using these prevention strategies relative to how it may affect their relationship and agreement.

By definition and in consideration of CIT, the process of establishing an SA could be advantageous for helping couples to reduce their risk for HIV and other STIs in several ways. First, it may provide couples with opportunities to learn and practice communication and negotiation skills, including the facilitation of discussions about their previous and current behaviors pertinent to prevention (eg, sex and substance use) and ways forward. Second, creation of an SA could help couples foster having a joint responsibility and identify ways for partners to support one another and for them to make associated decisions for how best to prevent HIV and other STIs in their relationship. For instance, creating an SA could enable couples with opportunities to decide if and when to use various evidence-based HIV/STI prevention strategies in their relationship according to their HIV serostatus and relational and sexual needs. Such strategies could include condom use, individual HIV/STI testing, [illegible]

CHTC, PrEP, and/or treatment as prevention (TasP) [41-44] with antiretroviral treatment (ART) to obtain and maintain an undetectable viral load to decrease the risk of onward HIV transmission among those living with HIV. As noted in prior work with male couples [45-51], the strategies which couples could include in their agreement may depend on the support and needs of each partner in the relationship, their attitudes toward these strategies, and their value and engagement in behaviors (eg, CAS and substance use) that may increase their risk for HIV yet be at odds with certain dynamics of their relationship (eg, trust and intimacy).

Couples-Based HIV Prevention Interventions for Male Couples

One meta-analysis has concluded that couples-based interventions are more effective in promoting sexual risk reduction behaviors and testing for HIV and other STIs when compared with interventions delivered to individual partners [8]. Although the evidence to support this conclusion is tempered by the limited number of efficacious HIV prevention interventions available for male couples [7], several theoretically informed, couples-based HIV prevention interventions have been developed for male couples [52-61], with several pending dissemination of outcome findings [52,53,56,57,60]. Many of these current and upcoming interventions use a tailored approach to accommodate couples' specific needs, incorporate communication and other dynamics in relationship skills–building activities (eg, problem solving), provide sexual health education and HIV/STI prevention-related resources, and encourage the formation of an SA or risk-reduction plan.

Several of the interventions include CHTC as one of the core components, either delivered in person [54,55,57,58] or remotely (ie, video Web-based platform) [53,56]. The number of sessions in the interventions vary, from 2 [52,53], 3 [57] and 4 [54,60] up to 7 sessions [61]. CHTC is a single-session intervention [58] that has also been pilot tested with an added component to address substance use [55]. The delivery time for these interventions also varies, ranging from 45 min for a single session (eg, CHTC) up to 10 or more hours for all sessions.

With respect to specific populations of male couples, 2 of the interventions were designed for young GBMSM in relationships [53,54]: one for methamphetamine-using black male couples [61] and another for predominantly Spanish-speaking Latino GBMSM and their same-sex partners [59,60]. Two of the interventions were developed to attend to the HIV care and adherence needs of male couples with one or both partners living with HIV (ie, serodiscordant and seroconcordant positive) [52,57], whereas some focus on the HIV prevention needs of seroconcordant negative and serodiscordant male couples [53,55,56,58]. Other interventions address the HIV prevention needs of all 3 groups of couples: seroconcordant negative, seroconcordant positive, and serodiscordant [54,60,61]. To date, 2 of the in-progress interventions are being delivered on the Web [53,56], whereas the rest are being or have been provided in person. In-person interventions for male couples may have limited impact and reachability because of structural barriers (eg, stigma of same-sex behaviors and lack of lesbian, gay, bisexual, transgender, and questioning/queer [LGBTQ]-affirming environments) and the number of resources (eg, appropriately trained personnel and cost) required for successful dissemination and implementation [62-66]. Interventions delivered by a digital health platform (ie, mobile health [mHealth] and electronic health [eHealth]) may help negate some of these limitations and required resources. Couples-based HIV prevention interventions that are delivered by a digital health platform would offer male couples the convenience of accessing the intervention from anywhere with an internet connection and being able to use it in a private setting, thereby providing further privacy, security, safety, and confidentiality. Pending the structure of the intervention, digital health platforms could also help increase reachability as more male couples would be able to use the intervention at any given time compared with those offered

couples would be able to use the intervention at any given time compared with those offered in person."

2b) In INTRODUCTION: Specific objectives or hypotheses

Does your paper address CONSORT subitem 2b? *

Copy and paste relevant sections from the manuscript (include quotes in quotation marks "like this" to indicate direct quotes from your manuscript), or elaborate on this item by providing additional information not in the ms, or briefly explain why the item is not applicable/relevant for your study

Yes, like this "The specific aims of the pilot randomized controlled trial (RCT) were to (1) assess the feasibility to recruit, enroll, and retain an eligible and consented sample of couples for 6 months; (2) assess the overall acceptability of the toolkit intervention; (3) examine the preliminary impact that using the toolkit intervention will result in a greater proportion of couples to establish and adhere to an SA compared with couples in the control condition; (4) describe the composition of couples' SAs relative to HIV/STI prevention; and (5) examine which relationship dynamics were associated with couples' establishment and/or adherence to an SA. The trial was not adequately powered to find meaningful differences between trial arms."

METHODS

3a) Description of trial design (such as parallel, factorial) including allocation ratio

Does your paper address CONSORT subitem 3a? *

Copy and paste relevant sections from the manuscript (include quotes in quotation marks "like this" to indicate direct quotes from your manuscript), or elaborate on this item by providing additional information not in the ms, or briefly explain why the item is not applicable/relevant for your study

Yes, like this "All procedures for the pilot RCT occurred on the Web, with couples randomly assigned to 1 of 2 conditions after completion of the baseline assessment. Figure 1 illustrates the Consolidated Standards of Reporting Trials diagram of the RCT.

All couples had to provide consent, pass eligibility and verification criteria, and post hoc validation tests to enroll into the pilot RCT. Through the electronic screener system, consented, eligible, and verified couples were then randomly assigned a unique enrollment ID containing a 4-digit, 2-letter combination that ended with either .01 or .02 to represent the specific partner in each relationship (eg, 1572SP.01 and 1572SP.02). A 4:4 block allocation was electronically generated and used to randomly assign couples' enrollment IDs to 1 of 2 eHealth conditions: an information-only control website or the interactive intervention website. Random assignment was double blinded; however, couples may have guessed which condition they were assigned once they completed the baseline assessment and were granted access to the rest of the eHealth website."

3b) Important changes to methods after trial commencement (such as eligibility criteria), with reasons

Does your paper address CONSORT subitem 3b? *

Copy and paste relevant sections from the manuscript (include quotes in quotation marks "like this" to indicate direct quotes from your manuscript), or elaborate on this item by providing additional information not in the ms, or briefly explain why the item is not applicable/relevant for your study

None - not applicable

3b-i) Bug fixes, Downtimes, Content Changes

Bug fixes, Downtimes, Content Changes: ehealth systems are often dynamic systems. A description of changes to methods therefore also includes important changes made on the intervention or comparator during the trial (e.g., major bug fixes or changes in the functionality or content) (5-iii) and other “unexpected events” that may have influenced study design such as staff changes, system failures/downtimes, etc. [2].

	1	2	3	4	5	
subitem not at all important	○	○	○	○	◉	essential

Does your paper address subitem 3b-i?

Copy and paste relevant sections from the manuscript (include quotes in quotation marks "like this" to indicate direct quotes from your manuscript), or elaborate on this item by providing additional information not in the ms, or briefly explain why the item is not applicable/relevant for your study

Not applicable - there weren't any fixes, downtimes or content changes.

4a) Eligibility criteria for participants

Does your paper address CONSORT subitem 4a? *

Copy and paste relevant sections from the manuscript (include quotes in quotation marks "like this" to indicate direct quotes from your manuscript), or elaborate on this item by providing additional information not in the ms, or briefly explain why the item is not applicable/relevant for your study

Yes, like this
"Eligibility Criteria
Each partner of the couple—independently—had to meet the following individual-level eligibility criteria to participate in the study: (1) self-report as cis-gender male, (2) aged at least 18 years, (3) be in a current sexual relationship with a main partner for 6 or more months, (4) self-report as HIV negative or unknown serostatus, (5) have had CAS with the primary partner within previous 6 months, (6) self-report no recent history of intimate partner violence or coercion within the previous year, (7) own a smartphone, and (8) have an alternate method to access the internet (eg, computer).
Couples with one or both partners who did not meet one or more of these criteria were individually ineligible for the study and were automatically informed after completion of the electronic screener. For instance, index partners who self-reported living with HIV received a message thanking them for their interest in the study and that they were ineligible to participate; because of being ineligible, his partner (ie, partner 2) would not have received a study invitation by email. The same ineligibility message was emailed to both partners of couples in instances where they were deemed ineligible and/or did not pass the relationship verification test (see the following sections). Thus, in addition to meeting the eligibility criteria, couples also had to pass the couple-level eligibility criteria through verification and validation tests to enroll for the pilot RCT.

Verification of Couples' Relationships and Validity of Their Data
After screener data were received from both partners, verification of the couples' relationship (ie, couple-level eligibility criteria) was done automatically through the electronic algorithm by evaluating and comparing each partner's response to 5 screener items and using predetermined decisions rules of acceptable responses (see Multimedia Appendix 1). Couples who received a score of 5 on 5 passed the verification test; all other scores were categorized as the couple not passing the verification test. Once a couple was deemed eligible with a verified relationship, we then manually conducted validity checks of their corresponding screener data on a case-by-case basis. Data validity checks consisted of evaluating the following information: repetition of same internet protocol (IP) address, use of suspicious participant name(s), presence of duplicate email or fictitious email addresses, back-to-back screener entries, presence of unique data responses to other screener items. For instance, back-to-back screener entries from the same IP address were permitted for a couple as long as all other benchmarks for validation passed. All other instances were flagged as fraudulent and were investigated further by contacting the potential participant/couple for clarification."

4a-i) Computer / Internet literacy

Computer / Internet literacy is often an implicit “de facto” eligibility criterion - this should be explicitly clarified.

	1	2	3	4	5	
subitem not at all important	○	○	◉	○	○	essential

Does your paper address subitem 4a-i?

Copy and paste relevant sections from the manuscript (include quotes in quotation marks "like this" to indicate direct quotes from your manuscript), or elaborate on this item by providing additional information not in the ms, or briefly explain why the item is not applicable/relevant for your study

Was more implied for eligibility criteria. Only item was "(8) have an alternate method to access the internet (eg, computer)."

4a-ii) Open vs. closed, web-based vs. face-to-face assessments:

Open vs. closed, web-based vs. face-to-face assessments: Mention how participants were recruited (online vs. offline), e.g., from an open access website or from a clinic, and clarify if this was a purely web-based trial, or there were face-to-face components (as part of the intervention or for assessment), i.e., to what degree got the study team to know the participant. In online-only trials, clarify if participants were quasi-anonymous and whether having multiple identities was possible or whether technical or logistical measures (e.g., cookies, email confirmation, phone calls) were used to detect/prevent these.

	1	2	3	4	5	
subitem not at all important	○	○	○	○	◉	essential

Does your paper address subitem 4a-ii? *

Copy and paste relevant sections from the manuscript (include quotes in quotation marks "like this" to indicate direct quotes from your manuscript), or elaborate on this item by providing additional information not in the ms, or briefly explain why the item is not applicable/relevant for your study

Like this "All procedures for the pilot RCT occurred on the Web....

... Recruitment and Screening Procedures
Targeted advertisements were placed on Facebook to recruit same-sex male couples over the course of 6 months;"

4a-iii) Information giving during recruitment

Information given during recruitment. Specify how participants were briefed for recruitment and in the informed consent procedures (e.g., publish the informed consent documentation as appendix, see also item X26), as this information may have an effect on user self-selection, user expectation and may also bias results.

	1	2	3	4	5	
subitem not at all important	(●)	( )	( )	( )	( )	essential

Does your paper address subitem 4a-iii?

Copy and paste relevant sections from the manuscript (include quotes in quotation marks "like this" to indicate direct quotes from your manuscript), or elaborate on this item by providing additional information not in the ms, or briefly explain why the item is not applicable/relevant for your study

Yes, like this "Each advertisement included a picture of a male couple with a brief study descriptor and a Web link that led interested individuals to the study introductory website. The study introductory website included webpages for the electronic consent document; eligibility screener; inputting and verifying contact information; and an embedded, electronic algorithm that automatically determined study eligibility at the individual and couple levels. The study introductory website was integrated with SurveyGizmo, a Health Insurance Portability and Accountability Act–compliant Web-based survey tool and database server, to collect and store data for the consent and eligibility screener."

4b) Settings and locations where the data were collected

Does your paper address CONSORT subitem 4b? *

Copy and paste relevant sections from the manuscript (include quotes in quotation marks "like this" to indicate direct quotes from your manuscript), or elaborate on this item by providing additional information not in the ms, or briefly explain why the item is not applicable/relevant for your study

Yes, like this "The advertisements targeted English-speaking adult males living in the United States (18 years) who had an interest in men and one of these relationship statuses: married, engaged, domestic partnership, civil union, or in a relationship."

4b-i) Report if outcomes were (self-)assessed through online questionnaires

Clearly report if outcomes were (self-)assessed through online questionnaires (as common in web-based trials) or otherwise.

	1	2	3	4	5	
subitem not at all important	○	○	○	○	◉	essential

Does your paper address subitem 4b-i? *

Copy and paste relevant sections from the manuscript (include quotes in quotation marks "like this" to indicate direct quotes from your manuscript), or elaborate on this item by providing additional information not in the ms, or briefly explain why the item is not applicable/relevant for your study

Yes, like this "All participants, regardless of the study arm, were asked to complete the 3-month and 6-month follow-up questionnaires.

The present analysis focuses on 2 outcomes: (1) establishment of an SA and (2) adherence to a SA. Data for these outcomes were collected at 3- and 6-month follow-up assessments from all participants, regardless of the study arm."

4b-ii) Report how institutional affiliations are displayed

Report how institutional affiliations are displayed to potential participants [on ehealth media], as affiliations with prestigious hospitals or universities may affect volunteer rates, use, and reactions with regards to an intervention.(Not a required item – describe only if this may bias results)

	1	2	3	4	5	
subitem not at all important	(●)	( )	( )	( )	( )	essential

Does your paper address subitem 4b-ii?

Copy and paste relevant sections from the manuscript (include quotes in quotation marks "like this" to indicate direct quotes from your manuscript), or elaborate on this item by providing additional information not in the ms, or briefly explain why the item is not applicable/relevant for your study

Institutional affiliations - often required by IRB - were displayed on consent. Not mentioned as this is common practice.

5) The interventions for each group with sufficient details to allow replication, including how and when they were actually administered

5-i) Mention names, credential, affiliations of the developers, sponsors, and owners

Mention names, credential, affiliations of the developers, sponsors, and owners [6] (if authors/evaluators are owners or developer of the software, this needs to be declared in a “Conflict of interest” section or mentioned elsewhere in the manuscript).

	1	2	3	4	5	
subitem not at all important	( )	( )	( )	( )	(●)	essential

Does your paper address subitem 5-i?

Copy and paste relevant sections from the manuscript (include quotes in quotation marks "like this" to indicate direct quotes from your manuscript), or elaborate on this item by providing additional information not in the ms, or briefly explain why the item is not applicable/relevant for your study

Yes, like this

"Description of the Electronic Health HIV Prevention Toolkit Intervention
The toolkit intervention involved participation at both individual and couple levels. At the individual level, participants experienced the interactive website in a directed, sequential fashion before being able to use the website with their relationship partner (ie, couple level). This dual-level intervention design was based on our formative work with 13 same-sex male couples who used the intervention as designed and provided feedback in focus groups (n=7 from Miami, FL, and n=6 from Atlanta, GA), whereas the content and design of the activities and videos were based from our qualitative findings with 29 couples from the metro areas of Detroit, MI, (n=13) and Atlanta, GA, (n=16) [20,22,39]. The vast majority of partnered men from these formative phases stated they wanted an opportunity to read the content, participate in the activities, and have time to digest the material before discussing and comparing their responses to the activities with their relationship partner, including the establish of an SA.
At the individual level, the intervention directed participants through a sequence of instructional and educational videos and modules about evidence-based HIV prevention strategies, communication tips, and SAs. In addition, 3 different activities were also embedded in this series of modules: the creation of a relationship timeline, identification and selection of relationship values, and establishment of an SA via a menu of options arranged by category (see the following sections). After completion of the baseline assessment, each participant was prompted to watch a brief, introductory, 1-min video about the purpose of toolkit intervention and how to use and navigate the website before proceeding to the relationship timeline and value instructional videos and activities. Next, participants were asked to read and review educational content about evidence-based HIV prevention strategies, followed by content on SAs, which included a video that offered suggestions of ways to bring up agreements in the relationship along with some common communication tips (eg, active listening). The last individual-level module was the agreement builder activity with an accompanying instructional video encouraging individuals to begin creating the SA they would like to have with their relationship partner.
Once both partners used the toolkit intervention as directed and added items to their agreement, they were then prompted (via text and email) to sign back into the toolkit intervention website as a couple. Using the toolkit intervention as a couple differed from when participants used it as individuals in important ways. First, the couple were shown their responses to the relationship timeline and value activities in a comparative fashion, which allowed partners to compare their responses and talk about where they differed and how they were similar. These activities served the purpose to prime partners to think about the fond memories they created (to date) and what they valued most about being in a relationship with one another collectively, before considering their future via an agreement. Then, the couple was shown a video about constructive communication tips (eg, negotiation) before proceeding to the agreement builder finalization activity. Similar to the other 2 activities, couples could also see—to a degree—how their individual selection of [illegible]

agreement items compared with one another as these pending items were arranged into 3 groupings: definitively wanted, potentially wanted with need to discuss, and did not want with discussion. Couples then negotiated which items they wanted to accept and place into their agreement or reject and place in the trash bin. Once all items were resolved, each partner would finalize his agreement by entering his unique password to the toolkit intervention.

Once a couple finalized their agreement, they could view all content, activities, and videos freely. Furthermore, the interactive website included a searchable resource center database (Sexual Health Resource Center) that allowed participants to find relevant sexual health resources in the United States and the option to download an app of a simplified version of the toolkit intervention that contained a blueprint of the couples finalized agreement, the ability to SMS/text within the relationship (ie, between partners), and the Sexual Health Resource Center. The educational content, videos, and activities were not available on the app.

HIV-Prevention Content

This educational module included text that described available evidence-based HIV prevention strategies, including female and male condoms, PrEP, nonoccupational postexposure prophylaxis, individual HIV/STI testing, and couple's HIV/STI testing.

Sexual Agreement Content

Another educational module focused on SAs. The content included an overarching definition of an SA along with different types of agreements that exist within the broader LGBTQ community (eg, closed, open with guidelines, and open without guidelines). Additional text drew from the extant literature about male couples' SAs to describe how common agreements are among male couples in the United States, what might motivate some to form an SA, the potential benefits of establishing an agreement in the relationship, whether agreements change over time, and the importance of communicating about the agreement in the relationship [17].

Relationship Timeline Activity

Participants could choose up to ten milestone life events that occurred throughout their relationship. Some examples of the events on the timeline activity included firsts such as first kiss, first time I met his family (or he met my family), first big purchase together. Each event was dated by the participant, which was then automatically placed chronologically in the visual format of a timeline.

Relationship Values Activity

Participants could choose up to 5 items that represented what they valued most about in a relationship with their current partner. Some examples of values presented in this activity were trusting each other, commitment to help our relationship grow, accepting our differences, feeling sexually satisfied with one another, and counting on each other.

Agreement Builder Activity

Participants could choose as many items as they wanted in their agreement. A total of 96 items were organized in 5 different categories: wellness (20 items; eg, testing for HIV, eating healthy, and supporting each other in our health goals), social etiquette (9 items; eg, holding hands with partner in public and having profiles on social media websites/apps), sex with my partner (23 items; eg, bottoming without condoms with partner, giving or getting head with partner, and group sex play as a couple), sex with other people (22 items; eg, topping with condoms with others, kissing with others, and sexting with others), and drugs (22 items; eg, alcohol with sex, ecstasy without sex, and erectile dysfunction medications). All 5 categories also included the option for participants to create their own and add details to each selected item.

Sexual Health Resource Center

Sexual Health Resource Center
This searchable database presented participants with contact and operational information about HIV/STI testing locations throughout the United States by zip code, testing modality preference (eg, individual, CHTC, and over the counter), appointment type (eg, walk-in and appointment required), and cost (eg, free and sliding fee). Locations of pharmacies were also included and searchable by zip code.
Information-Only Control Condition
Couples assigned to the information-only control condition also received an interactive website that contained the same HIV prevention content and Sexual Health Resource Center, along with access to download a similar app as the intervention group sans the blueprint of an agreement."

5-ii) Describe the history/development process

Describe the history/development process of the application and previous formative evaluations (e.g., focus groups, usability testing), as these will have an impact on adoption/use rates and help with interpreting results.

	1	2	3	4	5	
subitem not at all important	○	○	○	○	◉	essential

Does your paper address subitem 5-ii?

Copy and paste relevant sections from the manuscript (include quotes in quotation marks "like this" to indicate direct quotes from your manuscript), or elaborate on this item by providing additional information not in the ms, or briefly explain why the item is not applicable/relevant for your study

Yes, like this "The toolkit intervention involved participation at both individual and couple levels. At the individual level, participants experienced the interactive website in a directed, sequential fashion before being able to use the website with their relationship partner (ie, couple level). This dual-level intervention design was based on our formative work with 13 same-sex male couples who used the intervention as designed and provided feedback in focus groups (n=7 from Miami, FL, and n=6 from Atlanta, GA), whereas the content and design of the activities and videos were based from our qualitative findings with 29 couples from the metro areas of Detroit, MI, (n=13) and Atlanta, GA, (n=16) [20,22,39]. The vast majority of partnered men from these formative phases stated they wanted an opportunity to read the content, participate in the activities, and have time to digest the material before discussing and comparing their responses to the activities with their relationship partner, including the establish of an SA."

5-iii) Revisions and updating

Revisions and updating. Clearly mention the date and/or version number of the application/intervention (and comparator, if applicable) evaluated, or describe whether the intervention underwent major changes during the evaluation process, or whether the development and/or content was “frozen” during the trial. Describe dynamic components such as news feeds or changing content which may have an impact on the replicability of the intervention (for unexpected events see item 3b).

	1	2	3	4	5	
subitem not at all important	○	○	◉	○	○	essential

Does your paper address subitem 5-iii?

Copy and paste relevant sections from the manuscript (include quotes in quotation marks "like this" to indicate direct quotes from your manuscript), or elaborate on this item by providing additional information not in the ms, or briefly explain why the item is not applicable/relevant for your study

Not applicable as this did not happen - no changes (minor or major) were made during the pilot RCT.

5-iv) Quality assurance methods

Provide information on quality assurance methods to ensure accuracy and quality of information provided [1], if applicable.

	1	2	3	4	5	
subitem not at all important	○	○	◉	○	○	essential

Does your paper address subitem 5-iv?

Copy and paste relevant sections from the manuscript (include quotes in quotation marks "like this" to indicate direct quotes from your manuscript), or elaborate on this item by providing additional information not in the ms, or briefly explain why the item is not applicable/relevant for your study

Not applicable. Data were collected through self-assessments by participants.

5-v) Ensure replicability by publishing the source code, and/or providing screenshots/screen-capture video, and/or providing flowcharts of the algorithms used

Ensure replicability by publishing the source code, and/or providing screenshots/screen-capture video, and/or providing flowcharts of the algorithms used. Replicability (i.e., other researchers should in principle be able to replicate the study) is a hallmark of scientific reporting.

	1	2	3	4	5	
subitem not at all important	○	○	◉	○	○	essential

Does your paper address subitem 5-v?

Copy and paste relevant sections from the manuscript (include quotes in quotation marks "like this" to indicate direct quotes from your manuscript), or elaborate on this item by providing additional information not in the ms, or briefly explain why the item is not applicable/relevant for your study

Yes, like this
"Verification of Couples’ Relationships and Validity of Their Data
After screener data were received from both partners, verification of the couples’ relationship (ie, couple-level eligibility criteria) was done automatically through the electronic algorithm by evaluating and comparing each partner’s response to 5 screener items and using predetermined decisions rules of acceptable responses (see Multimedia Appendix 1). Couples who received a score of 5 on 5 passed the verification test; all other scores were categorized as the couple not passing the verification test. Once a couple was deemed eligible with a verified relationship, we then manually conducted validity checks of their corresponding screener data on a case-by-case basis. Data validity checks consisted of evaluating the following information: repetition of same internet protocol (IP) address, use of suspicious participant name(s), presence of duplicate email or fictitious email addresses, back-to-back screener entries, presence of unique data responses to other screener items. For instance, back-to-back screener entries from the same IP address were permitted for a couple as long as all other benchmarks for validation passed. All other instances were flagged as fraudulent and were investigated further by contacting the potential participant/couple for clarification."

5-vi) Digital preservation

Digital preservation: Provide the URL of the application, but as the intervention is likely to change or disappear over the course of the years; also make sure the intervention is archived (Internet Archive, webcitation.org, and/or publishing the source code or screenshots/videos alongside the article). As pages behind login screens cannot be archived, consider creating demo pages which are accessible without login.

	1	2	3	4	5	
subitem not at all important	○	○	●	○	○	essential

Does your paper address subitem 5-vi?

Copy and paste relevant sections from the manuscript (include quotes in quotation marks "like this" to indicate direct quotes from your manuscript), or elaborate on this item by providing additional information not in the ms, or briefly explain why the item is not applicable/relevant for your study

Not applicable as findings indicated the toolkit intervention needed to be updated/changed.

5-vii) Access

Access: Describe how participants accessed the application, in what setting/context, if they had to pay (or were paid) or not, whether they had to be a member of specific group. If known, describe how participants obtained “access to the platform and Internet” [1]. To ensure access for editors/reviewers/readers, consider to provide a “backdoor” login account or demo mode for reviewers/readers to explore the application (also important for archiving purposes, see vi).

	1	2	3	4	5	
subitem not at all important	○	○	○	○	●	essential

Does your paper address subitem 5-vii? *

Copy and paste relevant sections from the manuscript (include quotes in quotation marks "like this" to indicate direct quotes from your manuscript), or elaborate on this item by providing additional information not in the ms, or briefly explain why the item is not applicable/relevant for your study

Yes, participants were given a website with login information to create an account to access the Electronic Health HIV Prevention Toolkit Intervention. This information is mentioned in the Methods section.

5-viii) Mode of delivery, features/functionalities/components of the intervention and comparator, and the theoretical framework

Describe mode of delivery, features/functionalities/components of the intervention and comparator, and the theoretical framework [6] used to design them (instructional strategy [1], behaviour change techniques, persuasive features, etc., see e.g., [7, 8] for terminology). This includes an in-depth description of the content (including where it is coming from and who developed it) [1],” whether [and how] it is tailored to individual circumstances and allows users to track their progress and receive feedback” [6]. This also includes a description of communication delivery channels and – if computer-mediated communication is a component – whether communication was synchronous or asynchronous [6]. It also includes information on presentation strategies [1], including page design principles, average amount of text on pages, presence of hyperlinks to other resources, etc. [1].

	1	2	3	4	5	
subitem not at all important	○	○	○	○	◉	essential

Does your paper address subitem 5-viii? *

Copy and paste relevant sections from the manuscript (include quotes in quotation marks "like this" to indicate direct quotes from your manuscript), or elaborate on this item by providing additional information not in the ms, or briefly explain why the item is not applicable/relevant for your study

Yes, like this

"Couples Interdependence Theory
Couples interdependence theory (CIT) is a useful health behavior change theory to understand and examine the process in which relationship dynamics (ie, interaction between primary partners) positively and negatively impact male couples' decisions and behaviors relative to their risk for HIV and other STIs [11,12]. CIT describes one potential process of how relationship partners influence, initiate, and maintain behaviors that impact one another's health (ie, interdependence) [11,12]. Relative to HIV/STI prevention, this theoretical framework takes into consideration predisposing factors of the couple, which includes their relationship functioning (eg, commitment, satisfaction, and trust), communication style, perceptions of HIV/STIs as a health threat, and preferences for outcomes associated with the health threat (eg, condom use and testing).
In CIT, these predisposing factors are posited to affect couples' transformation of motivation and communal coping, 2 other key features of CIT. Transformation of motivation refers to the couple's cognitive interpretation and emotional response to the health threat as being meaningful (ie, important) to their relationship. In other words, partners move from a primarily individual-focused motivation to one that is more prorelationship and health enhancing (ie, how both partners as a couple benefit instead of only one) [11,12]. Transformation of motivation also lends itself to the couple, creating joint goals for long-term relationship functioning, and each partner's willingness to accommodate for the relationship is a function of the dynamics present in the relationship [13,14].
Another key component to CIT is communal coping, which refers to partners having a shared assessment of HIV/STIs as a health threat, a vision of shared action about managing and reducing their risk for HIV/STIs (via behaviors) and engaging in related HIV/STI prevention behaviors that are beneficial to them as a couple [11,12]. Couples' coping strategies for HIV/STI prevention are largely determined by the degree that both partners appraise HIV/STIs from an individual standpoint to one as a collective team (ie, transformation of motivation), such that their shared emotional and cognitive responses lead to a greater likelihood of them making a joint effort, partaking in planning and decision making and communicating about how best to reduce their risk for HIV and other STIs [15,16]. CIT provides a useful theoretical framework to examine how couples' dynamics in general and changes in their dynamics (eg, predisposing factors, transformation of motivation, and communal coping) may lead relationship partners to working together to engage in and achieve their joint health goals as it applies to HIV/STI prevention.

Description of the Electronic Health HIV Prevention Toolkit Intervention
The toolkit intervention involved participation at both individual and couple levels. At the individual level, participants experienced the interactive website in a directed, sequential fashion before being able to use the website with their relationship partner (ie, couple level). This dual-level intervention design was based on our formative work with 13 same-sex male couples who used the intervention as designed and provided feedback in focus groups (n=7 [illegible]

[illegible] from Miami, FL, and n=6 from Atlanta, GA), whereas the content and design of the activities and videos were based from our qualitative findings with 29 couples from the metro areas of Detroit, MI, (n=13) and Atlanta, GA, (n=16) [20,22,39]. The vast majority of partnered men from these formative phases stated they wanted an opportunity to read the content, participate in the activities, and have time to digest the material before discussing and comparing their responses to the activities with their relationship partner, including the establish of an SA.

At the individual level, the intervention directed participants through a sequence of instructional and educational videos and modules about evidence-based HIV prevention strategies, communication tips, and SAs. In addition, 3 different activities were also embedded in this series of modules: the creation of a relationship timeline, identification and selection of relationship values, and establishment of an SA via a menu of options arranged by category (see the following sections). After completion of the baseline assessment, each participant was prompted to watch a brief, introductory, 1-min video about the purpose of toolkit intervention and how to use and navigate the website before proceeding to the relationship timeline and value instructional videos and activities. Next, participants were asked to read and review educational content about evidence-based HIV prevention strategies, followed by content on SAs, which included a video that offered suggestions of ways to bring up agreements in the relationship along with some common communication tips (eg, active listening). The last individual-level module was the agreement builder activity with an accompanying instructional video encouraging individuals to begin creating the SA they would like to have with their relationship partner. Once both partners used the toolkit intervention as directed and added items to their agreement, they were then prompted (via text and email) to sign back into the toolkit intervention website as a couple. Using the toolkit intervention as a couple differed from when participants used it as individuals in important ways. First, the couple were shown their responses to the relationship timeline and value activities in a comparative fashion, which allowed partners to compare their responses and talk about where they differed and how they were similar. These activities served the purpose to prime partners to think about the fond memories they created (to date) and what they valued most about being in a relationship with one another collectively, before considering their future via an agreement. Then, the couple was shown a video about constructive communication tips (eg, negotiation) before proceeding to the agreement builder finalization activity. Similar to the other 2 activities, couples could also see—to a degree—how their individual selection of agreement items compared with one another as these pending items were arranged into 3 groupings: definitively wanted, potentially wanted with need to discuss, and did not want with discussion. Couples then negotiated which items they wanted to accept and place into their agreement or reject and place in the trash bin. Once all items were resolved, each partner would finalize his agreement by entering his unique password to the toolkit intervention.

Once a couple finalized their agreement, they could view all content, activities, and videos freely. Furthermore, the interactive website included a searchable resource center database (Sexual Health Resource Center) that allowed participants to find relevant sexual health resources in the United States and the option to download an app of a simplified version of the toolkit intervention that contained a blueprint of the couples finalized agreement, the ability to SMS/text within the relationship (ie, between partners), and the Sexual Health Resource Center. The educational content, videos, and activities were not available on the app.

HIV-Prevention Content

This educational module included text that described available evidence-based HIV [illegible]

This educational module included text that described available evidence-based HIV prevention strategies, including female and male condoms, PrEP, nonoccupational postexposure prophylaxis, individual HIV/STI testing, and couple's HIV/STI testing.
Sexual Agreement Content
Another educational module focused on SAs. The content included an overarching definition of an SA along with different types of agreements that exist within the broader LGBTQ community (eg, closed, open with guidelines, and open without guidelines). Additional text drew from the extant literature about male couples' SAs to describe how common agreements are among male couples in the United States, what might motivate some to form an SA, the potential benefits of establishing an agreement in the relationship, whether agreements change over time, and the importance of communicating about the agreement in the relationship [17].
Relationship Timeline Activity
Participants could choose up to ten milestone life events that occurred throughout their relationship. Some examples of the events on the timeline activity included firsts such as first kiss, first time I met his family (or he met my family), first big purchase together. Each event was dated by the participant, which was then automatically placed chronologically in the visual format of a timeline.
Relationship Values Activity
Participants could choose up to 5 items that represented what they valued most about in a relationship with their current partner. Some examples of values presented in this activity were trusting each other, commitment to help our relationship grow, accepting our differences, feeling sexually satisfied with one another, and counting on each other.
Agreement Builder Activity
Participants could choose as many items as they wanted in their agreement. A total of 96 items were organized in 5 different categories: wellness (20 items; eg, testing for HIV, eating healthy, and supporting each other in our health goals), social etiquette (9 items; eg, holding hands with partner in public and having profiles on social media websites/apps), sex with my partner (23 items; eg, bottoming without condoms with partner, giving or getting head with partner, and group sex play as a couple), sex with other people (22 items; eg, topping with condoms with others, kissing with others, and sexting with others), and drugs (22 items; eg, alcohol with sex, ecstasy without sex, and erectile dysfunction medications). All 5 categories also included the option for participants to create their own and add details to each selected item.
Sexual Health Resource Center
This searchable database presented participants with contact and operational information about HIV/STI testing locations throughout the United States by zip code, testing modality preference (eg, individual, CHTC, and over the counter), appointment type (eg, walk-in and appointment required), and cost (eg, free and sliding fee). Locations of pharmacies were also included and searchable by zip code.
Information-Only Control Condition
Couples assigned to the information-only control condition also received an interactive website that contained the same HIV prevention content and Sexual Health Resource Center, along with access to download a similar app as the intervention group sans the blueprint of an agreement."

5-ix) Describe use parameters

Describe use parameters (e.g., intended “doses” and optimal timing for use). Clarify what instructions or recommendations were given to the user, e.g., regarding timing, frequency, heaviness of use, if any, or was the intervention used ad libitum.

	1	2	3	4	5	
subitem not at all important	○	○	○	◉	○	essential

Does your paper address subitem 5-ix?

Copy and paste relevant sections from the manuscript (include quotes in quotation marks "like this" to indicate direct quotes from your manuscript), or elaborate on this item by providing additional information not in the ms, or briefly explain why the item is not applicable/relevant for your study

No - participants were not recommended to use a certain 'dose' of the intervention. They could use it as often as they wish during the 6 months.

5-x) Clarify the level of human involvement

Clarify the level of human involvement (care providers or health professionals, also technical assistance) in the e-intervention or as co-intervention (detail number and expertise of professionals involved, if any, as well as “type of assistance offered, the timing and frequency of the support, how it is initiated, and the medium by which the assistance is delivered”. It may be necessary to distinguish between the level of human involvement required for the trial, and the level of human involvement required for a routine application outside of a RCT setting (discuss under item 21 – generalizability).

	1	2	3	4	5	
subitem not at all important	○	○	◉	○	○	essential

Does your paper address subitem 5-x?

Copy and paste relevant sections from the manuscript (include quotes in quotation marks "like this" to indicate direct quotes from your manuscript), or elaborate on this item by providing additional information not in the ms, or briefly explain why the item is not applicable/relevant for your study

Not applicable. Intervention did not include any level of human involvement.

5-xi) Report any prompts/reminders used

Report any prompts/reminders used: Clarify if there were prompts (letters, emails, phone calls, SMS) to use the application, what triggered them, frequency etc. It may be necessary to distinguish between the level of prompts/reminders required for the trial, and the level of prompts/reminders for a routine application outside of a RCT setting (discuss under item 21 – generalizability).

	1	2	3	4	5	
subitem not at all important	○	○	○	◉	○	essential

Does your paper address subitem 5-xi? *

Copy and paste relevant sections from the manuscript (include quotes in quotation marks "like this" to indicate direct quotes from your manuscript), or elaborate on this item by providing additional information not in the ms, or briefly explain why the item is not applicable/relevant for your study

Yes, like this "Follow-up assessments occurred 3 and 6 months after baseline and took approximately 45 min to complete. Each participant was sent up to 2 reminders (email and text) about completing each follow-up assessment."

5-xii) Describe any co-interventions (incl. training/support)

Describe any co-interventions (incl. training/support): Clearly state any interventions that are provided in addition to the targeted eHealth intervention, as ehealth intervention may not be designed as stand-alone intervention. This includes training sessions and support [1]. It may be necessary to distinguish between the level of training required for the trial, and the level of training for a routine application outside of a RCT setting (discuss under item 21 – generalizability.

	1	2	3	4	5	
subitem not at all important	◉	○	○	○	○	essential

Does your paper address subitem 5-xii? *

Copy and paste relevant sections from the manuscript (include quotes in quotation marks "like this" to indicate direct quotes from your manuscript), or elaborate on this item by providing additional information not in the ms, or briefly explain why the item is not applicable/relevant for your study

Not applicable - no co-interventions were included.

6a) Completely defined pre-specified primary and secondary outcome measures, including how and when they were assessed

Does your paper address CONSORT subitem 6a? *

Copy and paste relevant sections from the manuscript (include quotes in quotation marks "like this" to indicate direct quotes from your manuscript), or elaborate on this item by providing additional information not in the ms, or briefly explain why the item is not applicable/relevant for your study

Yes, like this "The present analysis focuses on 2 outcomes: (1) establishment of an SA and (2) adherence to a SA. Data for these outcomes were collected at 3- and 6-month follow-up assessments from all participants, regardless of the study arm."

6a-i) Online questionnaires: describe if they were validated for online use and apply CHERRIES items to describe how the questionnaires were designed/deployed

If outcomes were obtained through online questionnaires, describe if they were validated for online use and apply CHERRIES items to describe how the questionnaires were designed/deployed [9].

	1	2	3	4	5	
subitem not at all important	○	○	●	○	○	essential

Does your paper address subitem 6a-i?

Copy and paste relevant sections from manuscript text

Yes, like this

"Measures
All participants, regardless of the study arm, were asked to complete the 3-month and 6-month follow-up questionnaires. The content of follow-up surveys matched the content of the baseline survey, except follow-up surveys also collected information on the formation of, type of, and adherence to an SA. In the event that couples ended their relationship, each partner was still asked to complete their participation throughout the 6 months to collect remaining data and receive their incentives. All data from baseline and follow-up assessments were deidentified, anonymized, and stored on secured servers and password-protected computers.

Outcome Variables
The present analysis focuses on 2 outcomes: (1) establishment of an SA and (2) adherence to a SA. Data for these outcomes were collected at 3- and 6-month follow-up assessments from all participants, regardless of the study arm.
Independent Variables
The baseline assessment captured participants' demographic (eg, state of residence, age, race, ethnicity, sexual orientation, education, employment, and health insurance regular primary provider) and relationship characteristics (eg, relationship length, type, status, and cohabitation) via categorical or dichotomous responses. A number of common sexual behavior items (eg, CAS by partner type) and measures about HIV/STI testing were also captured.
A variety of relationship dynamics were also assessed by using validated instruments for trust [67], relationship commitment [68], relationship satisfaction [69], relationship sexual satisfaction [70], intimacy [71], communication patterns [72], communal confidence [73], use of communal coping strategies to reduce HIV threat [73], preferences for sexual health outcomes [73], HIV social support scale [74], HIV-negative couples' perceptions of severity of HIV infection [73], investment in an SA [24], and preferences for general lifestyle outcomes [73]. Perceptions of local stigma [76], perceptions of gay-related stigma [73], and internalized homophobia [77] were also assessed. HIV-related dyadic measures developed for GBMSM in a relationship [73] offer a quantitative way to assess transformation of motivation and communal coping of CIT. All scales were assessed at all 3 time points (baseline, 3 months, and 6 months), except investment in an SA, which was assessed only at the 3- and 6-month time points. Details about the scales used to capture couples' relationship dynamics are provided in Multimedia Appendix 2."

6a-ii) Describe whether and how “use” (including intensity of use/dosage) was defined/measured/monitored

Describe whether and how “use” (including intensity of use/dosage) was defined/measured/monitored (logins, logfile analysis, etc.). Use/adoption metrics are important process outcomes that should be reported in any ehealth trial.

	1	2	3	4	5	
subitem not at all important	○	○	◉	○	○	essential

Does your paper address subitem 6a-ii?

Copy and paste relevant sections from manuscript text

Yes, like this

"Aim 2: Use and Acceptability of Toolkit Intervention
Over the period of 6 months, participants in the intervention arm logged into their eHealth toolkit an average of 13.42 times (range 1-38) compared with participants in the control arm who used their information-only website an average of 4.48 times (range 1-23). In total, 64.1% (191/298) participants downloaded the accompanying study app onto their smartphone: 65.4% (89/136) participants in the intervention arm (89 men representing 63 couples) and 63.0% (102/162) of participants in the control arm (102 men representing 74 couples). Differences were noted by arm with respect to whether one or both partners of the couple downloaded the app onto their smartphone. Specifically, a higher proportion of couples in the intervention arm (26/63 dyads, 41%) had both partners download the app compared with those in the control arm (28/74 dyads, 38%).
With respect to the acceptability of the eHealth HIV prevention toolkit, participants in the intervention arm also provided data about their perceptions of how easy it was to use various components of it, ranging from navigating the interactive website to using the agreement builder activity (Table 3). Participants reported, on average, that using different aspects of the intervention was easy for most items assessed across both time points. They also perceived downloading the accompanying smartphone app and using the Sexual Health Resource Center on the app was slightly less than easy, falling somewhere between neither difficult nor easy and easy across both time points."

6a-iii) Describe whether, how, and when qualitative feedback from participants was obtained

Describe whether, how, and when qualitative feedback from participants was obtained (e.g., through emails, feedback forms, interviews, focus groups).

	1	2	3	4	5	
subitem not at all important	○	○	●	○	○	essential

Does your paper address subitem 6a-iii?

Copy and paste relevant sections from manuscript text

Yes, in "Table 3. Acceptability data among participants in the intervention arm, by assessment time point."

6b) Any changes to trial outcomes after the trial commenced, with reasons

Does your paper address CONSORT subitem 6b? *

Copy and paste relevant sections from the manuscript (include quotes in quotation marks "like this" to indicate direct quotes from your manuscript), or elaborate on this item by providing additional information not in the ms, or briefly explain why the item is not applicable/relevant for your study

Not applicable. Trial outcomes remained the same.

7a) How sample size was determined

NPT: When applicable, details of whether and how the clustering by care provides or centers was addressed

7a-i) Describe whether and how expected attrition was taken into account when calculating the sample size

Describe whether and how expected attrition was taken into account when calculating the sample size.

	1	2	3	4	5	
subitem not at all important	(•)	( )	( )	( )	( )	essential

Does your paper address subitem 7a-i?

Copy and paste relevant sections from manuscript title (include quotes in quotation marks "like this" to indicate direct quotes from your manuscript), or elaborate on this item by providing additional information not in the ms, or briefly explain why the item is not applicable/relevant for your study

Not applicable as this was a pilot RCT to assess feasibility (to recruit, enroll, and retain participants/couples over time), acceptability (perceived ease of use of intervention), and preliminary impact of the intervention on couples' outcomes.

7b) When applicable, explanation of any interim analyses and stopping guidelines

Does your paper address CONSORT subitem 7b? *

Copy and paste relevant sections from the manuscript (include quotes in quotation marks "like this" to indicate direct quotes from your manuscript), or elaborate on this item by providing additional information not in the ms, or briefly explain why the item is not applicable/relevant for your study

Not applicable. All analyses were conducted once the pilot RCT closed.

8a) Method used to generate the random allocation sequence

NPT: When applicable, how care providers were allocated to each trial group

Does your paper address CONSORT subitem 8a? *

Copy and paste relevant sections from the manuscript (include quotes in quotation marks "like this" to indicate direct quotes from your manuscript), or elaborate on this item by providing additional information not in the ms, or briefly explain why the item is not applicable/relevant for your study

Yes, like this "A 4:4 block allocation was electronically generated and used to randomly assign couples' enrollment IDs to 1 of 2 eHealth conditions: an information-only control website or the interactive intervention website. Random assignment was double blinded; however, couples may have guessed which condition they were assigned once they completed the baseline assessment and were granted access to the rest of the eHealth website."

8b) Type of randomisation; details of any restriction (such as blocking and block size)

Does your paper address CONSORT subitem 8b? *

Copy and paste relevant sections from the manuscript (include quotes in quotation marks "like this" to indicate direct quotes from your manuscript), or elaborate on this item by providing additional information not in the ms, or briefly explain why the item is not applicable/relevant for your study

Yes, like this "A 4:4 block allocation was electronically generated and used to randomly assign couples' enrollment IDs to 1 of 2 eHealth conditions: an information-only control website or the interactive intervention website. Random assignment was double blinded; however, couples may have guessed which condition they were assigned once they completed the baseline assessment and were granted access to the rest of the eHealth website."

9) Mechanism used to implement the random allocation sequence (such as sequentially numbered containers), describing any steps taken to conceal the sequence until interventions were assigned

Does your paper address CONSORT subitem 9? *

Copy and paste relevant sections from the manuscript (include quotes in quotation marks "like this" to indicate direct quotes from your manuscript), or elaborate on this item by providing additional information not in the ms, or briefly explain why the item is not applicable/relevant for your study

Yes, like this "A 4:4 block allocation was electronically generated and used to randomly assign couples' enrollment IDs to 1 of 2 eHealth conditions: an information-only control website or the interactive intervention website. Random assignment was double blinded; however, couples may have guessed which condition they were assigned once they completed the baseline assessment and were granted access to the rest of the eHealth website."

10) Who generated the random allocation sequence, who enrolled participants, and who assigned participants to interventions

Does your paper address CONSORT subitem 10? *

Copy and paste relevant sections from the manuscript (include quotes in quotation marks "like this" to indicate direct quotes from your manuscript), or elaborate on this item by providing additional information not in the ms, or briefly explain why the item is not applicable/relevant for your study

The web developer.

11a) If done, who was blinded after assignment to interventions (for example, participants, care providers, those assessing outcomes) and how

NPT: Whether or not administering co-interventions were blinded to group assignment

11a-i) Specify who was blinded, and who wasn't

Specify who was blinded, and who wasn't. Usually, in web-based trials it is not possible to blind the participants [1, 3] (this should be clearly acknowledged), but it may be possible to blind outcome assessors, those doing data analysis or those administering co-interventions (if any).

	1	2	3	4	5	
subitem not at all important	○	○	◉	○	○	essential

Does your paper address subitem 11a-i? *

Copy and paste relevant sections from the manuscript (include quotes in quotation marks "like this" to indicate direct quotes from your manuscript), or elaborate on this item by providing additional information not in the ms, or briefly explain why the item is not applicable/relevant for your study

Yes, like this "A 4:4 block allocation was electronically generated and used to randomly assign couples' enrollment IDs to 1 of 2 eHealth conditions: an information-only control website or the interactive intervention website. Random assignment was double blinded; however, couples may have guessed which condition they were assigned once they completed the baseline assessment and were granted access to the rest of the eHealth website."

11a-ii) Discuss e.g., whether participants knew which intervention was the “intervention of interest” and which one was the “comparator”

Informed consent procedures (4a-ii) can create biases and certain expectations - discuss e.g., whether participants knew which intervention was the “intervention of interest” and which one was the “comparator”.

	1	2	3	4	5	
subitem not at all important	○	○	◉	○	○	essential

Does your paper address subitem 11a-ii?

Copy and paste relevant sections from the manuscript (include quotes in quotation marks "like this" to indicate direct quotes from your manuscript), or elaborate on this item by providing additional information not in the ms, or briefly explain why the item is not applicable/relevant for your study

Yes, like this "A 4:4 block allocation was electronically generated and used to randomly assign couples' enrollment IDs to 1 of 2 eHealth conditions: an information-only control website or the interactive intervention website. Random assignment was double blinded; however, couples may have guessed which condition they were assigned once they completed the baseline assessment and were granted access to the rest of the eHealth website."

11b) If relevant, description of the similarity of interventions

(this item is usually not relevant for ehealth trials as it refers to similarity of a placebo or sham intervention to a active medication/intervention)

Does your paper address CONSORT subitem 11b? *

Copy and paste relevant sections from the manuscript (include quotes in quotation marks "like this" to indicate direct quotes from your manuscript), or elaborate on this item by providing additional information not in the ms, or briefly explain why the item is not applicable/relevant for your study

Yes, like this "Information-Only Control Condition
Couples assigned to the information-only control condition also received an interactive website that contained the same HIV prevention content and Sexual Health Resource Center, along with access to download a similar app as the intervention group sans the blueprint of an agreement."

12a) Statistical methods used to compare groups for primary and secondary outcomes

NPT: When applicable, details of whether and how the clustering by care providers or centers was addressed

Does your paper address CONSORT subitem 12a? *

Copy and paste relevant sections from the manuscript (include quotes in quotation marks "like this" to indicate direct quotes from your manuscript), or elaborate on this item by providing additional information not in the ms, or briefly explain why the item is not applicable/relevant for your study

Yes, like this "Analyses
Descriptive statistics were used to summarize cohort characteristics and relationship dynamic variables for the entire cohort by trial arm and by establishment of SA. Dyadic data were calculated for couples if there were no missing values from either partner.
For continuous variables, couple-level mean variables were generated by taking the averaged value from both partners' scores, whereas within-dyad variables (couple-level differences) were generated by taking the absolute difference between 2 partners' scores. Missing values were assigned if either or both partners did not provide a score. Categorical dyadic variables were generated based on whether both partners had the same or different answers. For example, dyadic ethnicity was categorized to 3 levels: both Hispanic, 1 Hispanic, and neither Hispanic. Furthermore, 2-sample t tests and chi-square tests were used to evaluate differences between the intervention and control arms for couple-level continuous and categorical independent variables, respectively. To examine the association between relationship dynamics and establishment of an SA, we performed multilevel logistic regression analyses with random intercept for couples to account for correlations of repeated measurements of relationship dynamics at months 3 and 6 and reported the odds ratio (OR) of establishment of an SA and the corresponding 95% confidence interval. All analyses were performed using statistical software R 3.5.2."

12a-i) Imputation techniques to deal with attrition / missing values

Imputation techniques to deal with attrition / missing values: Not all participants will use the intervention/comparator as intended and attrition is typically high in ehealth trials. Specify how participants who did not use the application or dropped out from the trial were treated in the statistical analysis (a complete case analysis is strongly discouraged, and simple imputation techniques such as LOCF may also be problematic [4]).

	1	2	3	4	5	
subitem not at all important	○	○	●	○	○	essential

Does your paper address subitem 12a-i? *

Copy and paste relevant sections from the manuscript (include quotes in quotation marks "like this" to indicate direct quotes from your manuscript), or elaborate on this item by providing additional information not in the ms, or briefly explain why the item is not applicable/relevant for your study

Yes, like this "Descriptive statistics were used to summarize cohort characteristics and relationship dynamic variables for the entire cohort by trial arm and by establishment of SA. Dyadic data were calculated for couples if there were no missing values from either partner. For continuous variables, couple-level mean variables were generated by taking the averaged value from both partners' scores, whereas within-dyad variables (couple-level differences) were generated by taking the absolute difference between 2 partners' scores. Missing values were assigned if either or both partners did not provide a score. Categorical dyadic variables were generated based on whether both partners had the same or different answers. For example, dyadic ethnicity was categorized to 3 levels: both Hispanic, 1 Hispanic, and neither Hispanic. Furthermore, 2-sample t tests and chi-square tests were used to evaluate differences between the intervention and control arms for couple-level continuous and categorical independent variables, respectively. To examine the association between relationship dynamics and establishment of an SA, we performed multilevel logistic regression analyses with random intercept for couples to account for correlations of repeated measurements of relationship dynamics at months 3 and 6 and reported the odds ratio (OR) of establishment of an SA and the corresponding 95% confidence interval. All analyses were performed using statistical software R 3.5.2."

12b) Methods for additional analyses, such as subgroup analyses and adjusted analyses

Does your paper address CONSORT subitem 12b? *

Copy and paste relevant sections from the manuscript (include quotes in quotation marks "like this" to indicate direct quotes from your manuscript), or elaborate on this item by providing additional information not in the ms, or briefly explain why the item is not applicable/relevant for your study

not applicable; none were conducted.

X26) REB/IRB Approval and Ethical Considerations [recommended as subheading under "Methods"] (not a CONSORT item)

X26-i) Comment on ethics committee approval

	1	2	3	4	5	
subitem not at all important	○	○	○	○	◉	essential

Does your paper address subitem X26-i?

Copy and paste relevant sections from the manuscript (include quotes in quotation marks "like this" to indicate direct quotes from your manuscript), or elaborate on this item by providing additional information not in the ms, or briefly explain why the item is not applicable/relevant for your study

Yes, like this "The University of Miami's institutional review board approved all the study procedures. The pilot RCT was registered on ClinicalTrials.gov (NCT02494817)."

x26-ii) Outline informed consent procedures

Outline informed consent procedures e.g., if consent was obtained offline or online (how? Checkbox, etc.?), and what information was provided (see 4a-ii). See [6] for some items to be included in informed consent documents.

	1	2	3	4	5	
subitem not at all important	○	○	○	○	◉	essential

Does your paper address subitem X26-ii?

Copy and paste relevant sections from the manuscript (include quotes in quotation marks "like this" to indicate direct quotes from your manuscript), or elaborate on this item by providing additional information not in the ms, or briefly explain why the item is not applicable/relevant for your study

Yes, like this "The study introductory website included webpages for the electronic consent document; eligibility screener; inputting and verifying contact information; and an embedded, electronic algorithm that automatically determined study eligibility at the individual and couple levels. The study introductory website was integrated with SurveyGizmo, a Health Insurance Portability and Accountability Act–compliant Web-based survey tool and database server, to collect and store data for the consent and eligibility screener. On the basis of our prior work leading to this pilot RCT, the electronic algorithm—embedded within the Web-based screener—was developed and used to verify whether both partners of the couple were in a relationship with one another and had met all the eligibility criteria.
After providing consent and completing the screener for individual-level eligibility, potential participants (ie, index partner)..."

X26-iii) Safety and security procedures

Safety and security procedures, incl. privacy considerations, and any steps taken to reduce the likelihood or detection of harm (e.g., education and training, availability of a hotline)

	1	2	3	4	5	
subitem not at all important	○	○	◉	○	○	essential

Does your paper address subitem X26-iii?

Copy and paste relevant sections from the manuscript (include quotes in quotation marks "like this" to indicate direct quotes from your manuscript), or elaborate on this item by providing additional information not in the ms, or briefly explain why the item is not applicable/relevant for your study

Yes, like this ". The study introductory website included webpages for the electronic consent document; eligibility screener; inputting and verifying contact information; and an embedded, electronic algorithm that automatically determined study eligibility at the individual and couple levels. The study introductory website was integrated with SurveyGizmo, a Health Insurance Portability and Accountability Act–compliant Web-based survey tool and database server, to collect and store data for the consent and eligibility screener.

...

Couples with one or both partners who did not meet one or more of these criteria were individually ineligible for the study and were automatically informed after completion of the electronic screener."

RESULTS

13a) For each group, the numbers of participants who were randomly assigned, received intended treatment, and were analysed for the primary outcome

NPT: The number of care providers or centers performing the intervention in each group and the number of patients treated by each care provider in each center

Does your paper address CONSORT subitem 13a? *

Copy and paste relevant sections from the manuscript (include quotes in quotation marks "like this" to indicate direct quotes from your manuscript), or elaborate on this item by providing additional information not in the ms, or briefly explain why the item is not applicable/relevant for your study

Yes, like this "Of these 266 couples, 149 (56.0%) were enrolled in the pilot RCT as indicated in their creation of a required study profile and completion of the baseline assessment. Figure 2 shows retention rates for the 6-month pilot trial at the individual and couple levels. Overall, 71.5% (213/298) of partnered men were retained at the end of the 6-month pilot trial. Retention rates at the 3-month assessment were 67.6% (92/136) of partnered men in the intervention arm and 77.2% (125/162) of partnered men in the control arm (P=.07). Retention rates at the 6-month assessment were 72.1% (98/136) of partnered men in the intervention arm and 71.0% (115/162) of partnered men in the control arm (nonsignificant)."

13b) For each group, losses and exclusions after randomisation, together with reasons

Does your paper address CONSORT subitem 13b? (NOTE: Preferably, this is shown in a CONSORT flow diagram) *

Copy and paste relevant sections from the manuscript (include quotes in quotation marks "like this" to indicate direct quotes from your manuscript), or elaborate on this item by providing additional information not in the ms, or briefly explain why the item is not applicable/relevant for your study

Yes, like this

"As shown in Figure 1, 7959 individuals initiated screening resulting in 27.48% (2187/7959) of index partners being ineligible at the individual level; the remaining index partners were eligible at the individual level, but 18.04% (1436/7959) did not provide any contact information, 8.05% (641/7959) failed to verify their contact information, and 12.60% (1003/7959) did not have their partners (ie, partner 2) take the screener. The remaining screeners represented both partners of the couple, with 27.13% (2160/7959) being ineligible at the couple level among other reasons. Overall, 532 partners representing 266 couples passed the eligibility, verification, and validation screening process and were invited to participate in the pilot RCT via email invitation. Of these 266 couples, 149 (56.0%) were enrolled in the pilot RCT as indicated in their creation of a required study profile and completion of the baseline assessment.

Figure 2 shows retention rates for the 6-month pilot trial at the individual and couple levels. Overall, 71.5% (213/298) of partnered men were retained at the end of the 6-month pilot trial. Retention rates at the 3-month assessment were 67.6% (92/136) of partnered men in the intervention arm and 77.2% (125/162) of partnered men in the control arm (P=.07). Retention rates at the 6-month assessment were 72.1% (98/136) of partnered men in the intervention arm and 71.0% (115/162) of partnered men in the control arm (nonsignificant)."

13b-i) Attrition diagram

Strongly recommended: An attrition diagram (e.g., proportion of participants still logging in or using the intervention/comparator in each group plotted over time, similar to a survival curve) or other figures or tables demonstrating usage/dose/engagement.

	1	2	3	4	5	
subitem not at all important	○	○	●	○	○	essential

Does your paper address subitem 13b-i?

Copy and paste relevant sections from the manuscript or cite the figure number if applicable (include quotes in quotation marks "like this" to indicate direct quotes from your manuscript), or elaborate on this item by providing additional information not in the ms, or briefly explain why the item is not applicable/relevant for your study

Yes, like this

"Figure 2 shows retention rates for the 6-month pilot trial at the individual and couple levels. Overall, 71.5% (213/298) of partnered men were retained at the end of the 6-month pilot trial. Retention rates at the 3-month assessment were 67.6% (92/136) of partnered men in the intervention arm and 77.2% (125/162) of partnered men in the control arm (P=.07). Retention rates at the 6-month assessment were 72.1% (98/136) of partnered men in the intervention arm and 71.0% (115/162) of partnered men in the control arm (nonsignificant)."

14a) Dates defining the periods of recruitment and follow-up

Does your paper address CONSORT subitem 14a? *

Copy and paste relevant sections from the manuscript (include quotes in quotation marks "like this" to indicate direct quotes from your manuscript), or elaborate on this item by providing additional information not in the ms, or briefly explain why the item is not applicable/relevant for your study

Not for recruitment, but it does state for 6 month for recruitment and 6 months for follow-up. "Follow-up assessments occurred 3 and 6 months after baseline and took approximately 45 min to complete."

14a-i) Indicate if critical “secular events” fell into the study period

Indicate if critical “secular events” fell into the study period, e.g., significant changes in Internet resources available or “changes in computer hardware or Internet delivery resources”

	1	2	3	4	5	
subitem not at all important	○	○	●	○	○	essential

Does your paper address subitem 14a-i?

Copy and paste relevant sections from the manuscript (include quotes in quotation marks "like this" to indicate direct quotes from your manuscript), or elaborate on this item by providing additional information not in the ms, or briefly explain why the item is not applicable/relevant for your study

Unknown. Secular events may differ by individual regarding definition and meaning.

14b) Why the trial ended or was stopped (early)

Does your paper address CONSORT subitem 14b? *

Copy and paste relevant sections from the manuscript (include quotes in quotation marks "like this" to indicate direct quotes from your manuscript), or elaborate on this item by providing additional information not in the ms, or briefly explain why the item is not applicable/relevant for your study

Not applicable. It was not stopped early.

15) A table showing baseline demographic and clinical characteristics for each group

NPT: When applicable, a description of care providers (case volume, qualification, expertise, etc.) and centers (volume) in each group

Does your paper address CONSORT subitem 15? *

Copy and paste relevant sections from the manuscript (include quotes in quotation marks "like this" to indicate direct quotes from your manuscript), or elaborate on this item by providing additional information not in the ms, or briefly explain why the item is not applicable/relevant for your study

Yes, like this "Baseline characteristics and relationship dynamics for the total cohort and by study arm are provided in Tables 1 and 2, respectively."

15-i) Report demographics associated with digital divide issues

In ehealth trials it is particularly important to report demographics associated with digital divide issues, such as age, education, gender, social-economic status, computer/Internet/ehealth literacy of the participants, if known.

	1	2	3	4	5	
subitem not at all important	◉	○	○	○	○	essential

Does your paper address subitem 15-i? *

Copy and paste relevant sections from the manuscript (include quotes in quotation marks "like this" to indicate direct quotes from your manuscript), or elaborate on this item by providing additional information not in the ms, or briefly explain why the item is not applicable/relevant for your study

Not applicable due to eligibility criteria.

16) For each group, number of participants (denominator) included in each analysis and whether the analysis was by original assigned groups

16-i) Report multiple “denominators” and provide definitions

Report multiple “denominators” and provide definitions: Report N’s (and effect sizes) “across a range of study participation [and use] thresholds” [1], e.g., N exposed, N consented, N used more than x times, N used more than y weeks, N participants “used” the intervention/comparator at specific pre-defined time points of interest (in absolute and relative numbers per group). Always clearly define “use” of the intervention.

	1	2	3	4	5	
subitem not at all important	○	○	○	○	◉	essential

Does your paper address subitem 16-i? *

Copy and paste relevant sections from the manuscript (include quotes in quotation marks "like this" to indicate direct quotes from your manuscript), or elaborate on this item by providing additional information not in the ms, or briefly explain why the item is not applicable/relevant for your study

Yes in all figures, tables, and multimedia appendices.

16-ii) Primary analysis should be intent-to-treat

Primary analysis should be intent-to-treat, secondary analyses could include comparing only "users", with the appropriate caveats that this is no longer a randomized sample (see 18-i).

	1	2	3	4	5	
subitem not at all important	○	○	●	○	○	essential

Does your paper address subitem 16-ii?

Copy and paste relevant sections from the manuscript (include quotes in quotation marks "like this" to indicate direct quotes from your manuscript), or elaborate on this item by providing additional information not in the ms, or briefly explain why the item is not applicable/relevant for your study

Not applicable as outcomes required data be collected from both partners of the couple.

17a) For each primary and secondary outcome, results for each group, and the estimated effect size and its precision (such as 95% confidence interval)

Does your paper address CONSORT subitem 17a? *

Copy and paste relevant sections from the manuscript (include quotes in quotation marks "like this" to indicate direct quotes from your manuscript), or elaborate on this item by providing additional information not in the ms, or briefly explain why the item is not applicable/relevant for your study

Yes, in relevant Tables and Multimedia Appendices.

17a-i) Presentation of process outcomes such as metrics of use and intensity of use

In addition to primary/secondary (clinical) outcomes, the presentation of process outcomes such as metrics of use and intensity of use (dose, exposure) and their operational definitions is critical. This does not only refer to metrics of attrition (13-b) (often a binary variable), but also to more continuous exposure metrics such as "average session length". These must be accompanied by a technical description how a metric like a "session" is defined (e.g., timeout after idle time) [1] (report under item 6a).

	1	2	3	4	5	
subitem not at all important	○	○	◉	○	○	essential

Does your paper address subitem 17a-i?

Copy and paste relevant sections from the manuscript (include quotes in quotation marks "like this" to indicate direct quotes from your manuscript), or elaborate on this item by providing additional information not in the ms, or briefly explain why the item is not applicable/relevant for your study

Yes, like this "Over the period of 6 months, participants in the intervention arm logged into their eHealth toolkit an average of 13.42 times (range 1-38) compared with participants in the control arm who used their information-only website an average of 4.48 times (range 1-23). In total, 64.1% (191/298) participants downloaded the accompanying study app onto their smartphone: 65.4% (89/136) participants in the intervention arm (89 men representing 63 couples) and 63.0% (102/162) of participants in the control arm (102 men representing 74 couples). Differences were noted by arm with respect to whether one or both partners of the couple downloaded the app onto their smartphone. Specifically, a higher proportion of couples in the intervention arm (26/63 dyads, 41%) had both partners download the app compared with those in the control arm (28/74 dyads, 38%)."

17b) For binary outcomes, presentation of both absolute and relative effect sizes is recommended

Does your paper address CONSORT subitem 17b? *

Copy and paste relevant sections from the manuscript (include quotes in quotation marks "like this" to indicate direct quotes from your manuscript), or elaborate on this item by providing additional information not in the ms, or briefly explain why the item is not applicable/relevant for your study

Yes, in relevant Tables and Multimedia Appendices.

18) Results of any other analyses performed, including subgroup analyses and adjusted analyses, distinguishing pre-specified from exploratory

Does your paper address CONSORT subitem 18? *

Copy and paste relevant sections from the manuscript (include quotes in quotation marks "like this" to indicate direct quotes from your manuscript), or elaborate on this item by providing additional information not in the ms, or briefly explain why the item is not applicable/relevant for your study

Was not performed; not applicable.

18-i) Subgroup analysis of comparing only users

A subgroup analysis of comparing only users is not uncommon in ehealth trials, but if done, it must be stressed that this is a self-selected sample and no longer an unbiased sample from a randomized trial (see 16-iii).

	1	2	3	4	5	
subitem not at all important	○	○	◉	○	○	essential

Does your paper address subitem 18-i?

Copy and paste relevant sections from the manuscript (include quotes in quotation marks "like this" to indicate direct quotes from your manuscript), or elaborate on this item by providing additional information not in the ms, or briefly explain why the item is not applicable/relevant for your study

Not applicable/relevant due to nature of outcomes being dyadic, couple-level.

19) All important harms or unintended effects in each group

(for specific guidance see CONSORT for harms)

Does your paper address CONSORT subitem 19? *

Copy and paste relevant sections from the manuscript (include quotes in quotation marks "like this" to indicate direct quotes from your manuscript), or elaborate on this item by providing additional information not in the ms, or briefly explain why the item is not applicable/relevant for your study

None happened, none reported.

19-i) Include privacy breaches, technical problems

Include privacy breaches, technical problems. This does not only include physical “harm” to participants, but also incidents such as perceived or real privacy breaches [1], technical problems, and other unexpected/unintended incidents. “Unintended effects” also includes unintended positive effects [2].

	1	2	3	4	5	
subitem not at all important	○	○	●	○	○	essential

Does your paper address subitem 19-i?

Copy and paste relevant sections from the manuscript (include quotes in quotation marks "like this" to indicate direct quotes from your manuscript), or elaborate on this item by providing additional information not in the ms, or briefly explain why the item is not applicable/relevant for your study

None happened, none reported.

19-ii) Include qualitative feedback from participants or observations from staff/researchers

Include qualitative feedback from participants or observations from staff/researchers, if available, on strengths and shortcomings of the application, especially if they point to unintended/unexpected effects or uses. This includes (if available) reasons for why people did or did not use the application as intended by the developers.

	1	2	3	4	5	
subitem not at all important	○	○	◉	○	○	essential

Does your paper address subitem 19-ii?

Copy and paste relevant sections from the manuscript (include quotes in quotation marks "like this" to indicate direct quotes from your manuscript), or elaborate on this item by providing additional information not in the ms, or briefly explain why the item is not applicable/relevant for your study

Yes, provided in Table 3.

DISCUSSION

22) Interpretation consistent with results, balancing benefits and harms, and considering other relevant evidence

NPT: In addition, take into account the choice of the comparator, lack of or partial blinding, and unequal expertise of care providers or centers in each group

22-i) Restate study questions and summarize the answers suggested by the data, starting with primary outcomes and process outcomes (use)

Restate study questions and summarize the answers suggested by the data, starting with primary outcomes and process outcomes (use).

	1	2	3	4	5	
subitem not at all important	○	○	○	○	◉	essential

Does your paper address subitem 22-i? *

Copy and paste relevant sections from the manuscript (include quotes in quotation marks "like this" to indicate direct quotes from your manuscript), or elaborate on this item by providing additional information not in the ms, or briefly explain why the item is not applicable/relevant for your study

Yes, like this "The findings from this pilot RCT suggest the feasibility and acceptability of an eHealth HIV prevention toolkit intervention to encourage establishment and adherence to an SA among seroconcordant negative male couples.

Acceptability and Use
Overall, participants reported high acceptability of the toolkit intervention. This was particularly true for navigating and using the different components of the interactive website and for individually selecting and then negotiating and finalizing the creation of an SA with their relationship partner. Their acceptability was slightly lower for downloading the accompanying smartphone app and using the Sexual Health Resource Center on the app. It is possible that participants may have had connectivity issues in downloading and/or while using the app, thereby influencing their attitudes toward this part of the toolkit. It is also possible that participants may have perceived the app to be too simplistic and questioned the need for it given the stark contrast of what the app offered compared with the interactive website. Moreover, the items used to assess participants' acceptability of this couples-based intervention may not have captured all key elements and/or their attitudes about it. Future digital health, couples-based interventions may want to consider using the Health Information Technology Usability Evaluation Scale, a customizable usability evaluation instrument that includes subscales of impact, perceived usefulness, ease of use, and user control [80].
Furthermore, a future iteration of this couples-based HIV/STI prevention intervention toolkit may need to be offered in a variety of formats to further increase reach, access, and acceptability among the target population. It is possible that some of the eligible and consented couples who chose not to enroll (eg, 117/266), did so because they realized the intervention could only be used on a laptop of desktop computer as it was not optimized for smaller screen devices. Moving forward, the toolkit may need to be delivered on a single responsive website that would work across all types of internet-connected devices, including smartphones, tablets, laptops, and desktop computers. It could also be offered on an app for smartphones and tablets, which would allow researchers to send reminders and notifications directly to participants (eg, time to complete an assessment). Future research with the target population is warranted to help decide whether one or both of these options for the next iteration of the toolkit ought to be offered.
Acceptability of the agreement builder activity must also be considered. Overall, participants liked the agreement builder activity and how they experienced and used it (solo followed by as a couple). They also provided feedback about how often they thought they would use it over time. About one-third of the participants thought they would use this activity on a regular basis (ie, at some interval), one-third of them perceived they would use it on an as-needed basis, and one-quarter of them were unsure; few of them said they would never use this type of activity. Similar to the importance of being tested for HIV/STIs at a regular interval (eg, every 3, 6, or 12 months), we believe using the agreement builder activity at a regular interval would be beneficial for the couple. SAs are fluid and could change over time to reflect partners' and couples' evolving needs. This type of activity would allow couples to [illegible]

revisit and change their agreement, and it would also provide couples with opportunities to help improve their understanding about behaviors they wish to agree to engage in and not engage in (ie, within-couple concordance). Findings from a recent study with male couples from Boston, Atlanta, and Chicago support this idea. The authors reported weak-to-moderate concordance on couples' agreements guidelines that pertained to having sex outside of the relationship and for specific sexual behaviors they allowed or disallowed to occur [30]. Although we do not think couples ought to be forced into these types of conversations, a toolkit could be programmed to periodically check in with each partner of the couple to assess their overall satisfaction with the agreement and whether their sexual health and relationship needs have changed from when they first created their agreement or from their last check-in. A future version of this activity could provide this kind of check-in mechanism, either preprogrammed or by a time interval (eg, quarterly) set by both partners of the couple.

Participants mentioned another area of the SA builder activity that warrants attention. Some perceived the agreement builder activity contained too many items for them to consider for their SA (Table 3). In addition, approximately one-quarter to one-third of couples included HIV/STI prevention items in their SA (Table 5), and 28% specified whether sex was permitted with casual GBMSM partners. In its current form, the agreement builder activity enabled couples to choose and select items for their SA from a menu consisting of 5 categories with a total of 96 items. This approach, although deemed to be acceptable in our formative work leading to the pilot trial, may have diminished the focus on HIV/STI prevention and overwhelmed some of the partners/couples given the array of choices. It is also possible that some of the couples may have perceived their risk for HIV/STIs to be low and opted to not include any items about prevention. Prior research has found that couples perceived their risk for HIV and other STIs to be generally low, in part because of their beliefs that being in a relationship—by virtue—incorrectly reduces their risk or protects them from HIV/STIs [81]. One possible solution to encourage couples to include HIV/STI prevention items in their SA is to restructure, streamline, and simplify the agreement builder activity. First, an electronic algorithm could be embedded in the activity to prompt each partner of the couple to answer a brief set of questions to gauge the kind of sexual relationship they would want and the types of sexual behaviors they would prefer to engage in. Their responses to these questions could then automatically generate and place HIV/STI prevention items in their agreement for a more directed approach. Furthermore, the agreement builder activity could be broken down into several segments for couples to complete over time and not in one sitting. For example, once a couple decides which HIV/STI prevention items to include in their SA, they could then be prompted to revisit the agreement builder activity to focus on a different area that they deem to be important, such as strengthening and affirming their relationship. Changing the agreement builder activity is these ways (ie, algorithm, directed, and staggered) may help encourage couples to use the toolkit over time and simplify the process of building an agreement that meets their prevention and relationship needs (while lessening their feelings of being overwhelmed by too many choices).

Sexual Agreement Outcomes

The preliminary impact of the eHealth HIV prevention toolkit intervention on couples' establishment and adherence to an SA was also assessed. Compared with couples in the control arm, more couples in the intervention arm established an SA over time. Although a significant difference for establishing an SA was found at the 6-month follow-up between the 2 trial arms, the pilot trial was not adequately powered as we were more interested in obtaining point estimates and trends. These findings show initial promise for the toolkit intervention to help encourage couples who did not have an SA to establish one. However

intervention to help encourage couples who did not have an SA to establish one. However, there may be other possibilities that influenced couples to establish an SA, either apart (for couples in either trial arm) or in addition to using the toolkit (intervention arm only). Prior research has described that for some couples, certain circumstances or experiences (eg, events with others and influences from peers) may have led them to forming an SA [22]. It is also possible that couples established an SA as part of their natural progression in the relationship [19,33] and to enhance or improve an aspect of their relationship (eg, trust and intimacy) [21]. Future couples-based research that includes the establishment of an SA in the intervention would benefit to include an evaluation item to assess what influenced couples to form an agreement in their relationship.

A number of common relationship dynamics (eg, constructive communication, intimacy, and communal coping strategies to reduce HIV threat) at the averaged couple level were positively associated with couples establishing an SA—in general and over time. Similar findings were noted for lower averaged partner score differences being positively associated with couples establishing an SA. These findings align with what prior research with male couples has highlighted [6,26,82,83]: including and bolstering relationship dynamics along with sexual identity affirmation in couples-based interventions is critically important for HIV/STI prevention. It should be noted that findings from this trial suggest men's perceptions about how much stigma there is for being gay in their local community and for being in a same-sex relationship may play an important role in HIV/STI prevention with male couples by decreasing their odds of establishing an SA. Specifically, as scores of the averaged couple level and differences between partners increase for these measures, the odds of a couple establishing an agreement decrease between 65% and 79%. Limited research has investigated the role that male couples' living and social environment(s) may have toward their risk for HIV/STIs [82,84,85], particularly with respect to internalized and perceived stigma. Further research is warranted to examine the ways in which stigma may impact male couples' relationships and efforts related to HIV/STI prevention.

With respect to adherence, fewer couples in the intervention arm broke their SA over time compared with couples in the control arm. Differences between the 2 trial arms were nonsignificant for both follow-up time points. Sample size constraints prevented our ability to quantitatively assess and meaningfully detect whether any differences in relationship dynamics existed between couples who broke their agreement compared with those who adhered to their agreement. A future trial with a larger sample size and longer follow-up time period (eg, 12 or 18 months) may provide a greater likelihood to assess any differences between couples who adhered to and did not adhere to their agreement, as had been found in a recent longitudinal study with male couples [86]. In addition, nonadherence to an SA may be defined differently between partners of the couple, which could influence how they might report about it. Recent research with male couples has found partner's reports on what components and behaviors their agreement included did not always align [17,30], which could in turn affect their understanding of the agreement and their report of adherence. As such, better measurements are needed to improve detection of agreement breaks by considering the different components (eg, emotional and sexual) of a couples' agreement.
"

22-ii) Highlight unanswered new questions, suggest future research

Highlight unanswered new questions, suggest future research.

	1	2	3	4	5	
subitem not at all important	○	○	○	○	◉	essential

Does your paper address subitem 22-ii?

Copy and paste relevant sections from the manuscript (include quotes in quotation marks "like this" to indicate direct quotes from your manuscript), or elaborate on this item by providing additional information not in the ms, or briefly explain why the item is not applicable/relevant for your study

Yes, like this "The study also did not include serodiscordant and seroconcordant positive male couples or partnered transgender individuals (eg, transmen)—other populations who are in need of accessible, couples-based HIV/STI prevention interventions. A future iteration of the toolkit should include the biomedical (eg, Undetectable=Untransmissible and TasP), behavioral, and relational needs of serodiscordant and seroconcordant positive male couples [87] and transgender individuals and their relationship partners."

20) Trial limitations, addressing sources of potential bias, imprecision, and, if relevant, multiplicity of analyses

20-i) Typical limitations in ehealth trials

Typical limitations in ehealth trials: Participants in ehealth trials are rarely blinded. Ehealth trials often look at a multiplicity of outcomes, increasing risk for a Type I error. Discuss biases due to non-use of the intervention/usability issues, biases through informed consent procedures, unexpected events.

	1	2	3	4	5	
subitem not at all important	○	○	○	○	◉	essential

Does your paper address subitem 20-i? *

Copy and paste relevant sections from the manuscript (include quotes in quotation marks "like this" to indicate direct quotes from your manuscript), or elaborate on this item by providing additional information not in the ms, or briefly explain why the item is not applicable/relevant for your study

Yes, like this
"Limitations
This pilot RCT has several limitations. A convenience sample was recruited by placement of targeted advertisements on Facebook, thereby limiting the generalizability of the study's findings as not all partnered men may use Facebook and those who do may not respond to advertisements about participating in HIV prevention or relationship research studies. Second, establishment and adherence to an SA were assessed by self-reporting. Social desirability bias may have influenced participants' responses to these survey items, thereby potentially affecting the study's outcome findings. The study also did not include serodiscordant and seroconcordant positive male couples or partnered transgender individuals (eg, transmen)—other populations who are in need of accessible, couples-based HIV/STI prevention interventions. A future iteration of the toolkit should include the biomedical (eg, Undetectable=Untransmissible and TasP), behavioral, and relational needs of serodiscordant and seroconcordant positive male couples [87] and transgender individuals and their relationship partners. Despite these limitations, findings from this pilot study showed promise for encouraging couples to establish and adhere to their SAs to warrant continuation of this research for HIV/STI prevention. A future trial of the updated toolkit with a larger sample size would provide sufficient power to detect effects and changes over time to assess whether establishing and adhering to an SA could enhance HIV/STI prevention efforts for male couples."

21) Generalisability (external validity, applicability) of the trial findings

NPT: External validity of the trial findings according to the intervention, comparators, patients, and care providers or centers involved in the trial

21-i) Generalizability to other populations

Generalizability to other populations: In particular, discuss generalizability to a general Internet population, outside of a RCT setting, and general patient population, including applicability of the study results for other organizations

	1	2	3	4	5	
subitem not at all important	○	○	○	●	○	essential

Does your paper address subitem 21-i?

Copy and paste relevant sections from the manuscript (include quotes in quotation marks "like this" to indicate direct quotes from your manuscript), or elaborate on this item by providing additional information not in the ms, or briefly explain why the item is not applicable/relevant for your study

Yes, like this "A convenience sample was recruited by placement of targeted advertisements on Facebook, thereby limiting the generalizability of the study's findings as not all partnered men may use Facebook and those who do may not respond to advertisements about participating in HIV prevention or relationship research studies. Second, establishment and adherence to an SA were assessed by self-reporting. Social desirability bias may have influenced participants' responses to these survey items, thereby potentially affecting the study's outcome findings."

21-ii) Discuss if there were elements in the RCT that would be different in a routine application setting

Discuss if there were elements in the RCT that would be different in a routine application setting (e.g., prompts/reminders, more human involvement, training sessions or other co-interventions) and what impact the omission of these elements could have on use, adoption, or outcomes if the intervention is applied outside of a RCT setting.

	1	2	3	4	5	
subitem not at all important	(selected)	○	○	○	○	essential

Does your paper address subitem 21-ii?

Copy and paste relevant sections from the manuscript (include quotes in quotation marks "like this" to indicate direct quotes from your manuscript), or elaborate on this item by providing additional information not in the ms, or briefly explain why the item is not applicable/relevant for your study

Not applicable as this was intended to be an Electronic Health Intervention and not one for in-person delivery.

OTHER INFORMATION

23) Registration number and name of trial registry

Does your paper address CONSORT subitem 23? *

Copy and paste relevant sections from the manuscript (include quotes in quotation marks "like this" to indicate direct quotes from your manuscript), or elaborate on this item by providing additional information not in the ms, or briefly explain why the item is not applicable/relevant for your study

Yes, like this "The pilot RCT was registered on ClinicalTrials.gov (NCT02494817)."

24) Where the full trial protocol can be accessed, if available

Does your paper address CONSORT subitem 24? *

Cite a Multimedia Appendix, other reference, or copy and paste relevant sections from the manuscript (include quotes in quotation marks "like this" to indicate direct quotes from your manuscript), or elaborate on this item by providing additional information not in the ms, or briefly explain why the item is not applicable/relevant for your study

Yes, like this "The pilot RCT was registered on ClinicalTrials.gov (NCT02494817)." And through this publication.

25) Sources of funding and other support (such as supply of drugs), role of funders

Does your paper address CONSORT subitem 25? *

Copy and paste relevant sections from the manuscript (include quotes in quotation marks "like this" to indicate direct quotes from your manuscript), or elaborate on this item by providing additional information not in the ms, or briefly explain why the item is not applicable/relevant for your study

Yes, like this "This work was supported by the National Institute of Mental Health under Grant R34 MH102098 (principal investigator JM)."

X27) Conflicts of Interest (not a CONSORT item)

X27-i) State the relation of the study team towards the system being evaluated

In addition to the usual declaration of interests (financial or otherwise), also state the relation of the study team towards the system being evaluated, i.e., state if the authors/evaluators are distinct from or identical with the developers/sponsors of the intervention.

	1	2	3	4	5	
subitem not at all important	○	○	○	○	●	essential

Does your paper address subitem X27-i?

Copy and paste relevant sections from the manuscript (include quotes in quotation marks "like this" to indicate direct quotes from your manuscript), or elaborate on this item by providing additional information not in the ms, or briefly explain why the item is not applicable/relevant for your study

Yes, like this
"Conflicts of Interest
None declared."

About the CONSORT EHEALTH checklist

As a result of using this checklist, did you make changes in your manuscript? *

- ○ yes, major changes
- ○ yes, minor changes
- ● no

What were the most important changes you made as a result of using this checklist?

Not applicable.

How much time did you spend on going through the checklist INCLUDING making changes in your manuscript *

60 minutes

As a result of using this checklist, do you think your manuscript has improved? *

- ○ yes
- ◉ no
- ○ Other:

Would you like to become involved in the CONSORT EHEALTH group?

This would involve for example becoming involved in participating in a workshop and writing an "Explanation and Elaboration" document

- ◉ yes
- ○ no
- ○ Other:

Any other comments or questions on CONSORT EHEALTH

Your answer

STOP - Save this form as PDF before you click submit

To generate a record that you filled in this form, we recommend to generate a PDF of this page (on a Mac, simply select "print" and then select "print as PDF") before you submit it.

When you submit your (revised) paper to JMIR, please upload the PDF as supplementary file.

Don't worry if some text in the textboxes is cut off, as we still have the complete information in our database. Thank you!

Final step: Click submit !

Click submit so we have your answers in our database!

Submit

Never submit passwords through Google Forms.

This form was created outside of your domain. Report Abuse - Terms of Service - Privacy Policy

Google Forms
